# New Approaches and Understandings in the Growth of Cubic Silicon Carbide

**DOI:** 10.3390/ma14185348

**Published:** 2021-09-16

**Authors:** Francesco La Via, Massimo Zimbone, Corrado Bongiorno, Antonino La Magna, Giuseppe Fisicaro, Ioannis Deretzis, Viviana Scuderi, Cristiano Calabretta, Filippo Giannazzo, Marcin Zielinski, Ruggero Anzalone, Marco Mauceri, Danilo Crippa, Emilio Scalise, Anna Marzegalli, Andrey Sarikov, Leo Miglio, Valdas Jokubavicius, Mikael Syväjärvi, Rositsa Yakimova, Philipp Schuh, Michael Schöler, Manuel Kollmuss, Peter Wellmann

**Affiliations:** 1 Consiglio Nazionale delle Ricerche – Istituto per la Microelettronice e Microsistemi, Strada VIII 5, 95121 Catania, Italy; massimo.zimbone@imm.cnr.it (M.Z.); corrado.bongiorno@imm.cnr.it (C.B.); antonino.lamagna@imm.cnr.it (A.L.M.); Giuseppe.fisicaro@imm.cnr.it (G.F.); ioannis.deretzis@imm.cnr.it (I.D.); viviana.scuderi@imm.cnr.it (V.S.); cristiano.calabretta@imm.cnr.it (C.C.); filippo.giannazzo@imm.cnr.it (F.G.); 2 NOVASIC, Savoie Technolac—Arche Bat.4, Allée du Lac d’Aiguebelette, BP 267, 73375 Le Bourget du Lac CEDEX, France; mzielinski@novasic.com; 3 STMicroelectronics, Stradale Primosole 50, 95121 Catania, Italy; ruggero.anzalone@st.com; 4 LPE, Strada XVI, Pantano d’Arci, 95121 Catania, Italy; Marco.Mauceri@lpe-epi.com (M.M.); danilo.crippa@lpe-epi.com (D.C.); 5 L-NESS and Department of Materials Science, Università di Milano-Bicocca, Via R. Cozzi 55, I-20125 Milano, Italy; emilio.scalise@unimib.it (E.S.); andrey.sarikov@unimib.it (A.S.); leo.miglio@unimib.it (L.M.); 6 L-NESS and Department of Physics, Politecnico di Milano, via Anzani 42, 22100 Como, Italy; anna.marzegalli@unimib.it; 7 Department of Physics, Chemistry, and Biology (IFM), Linköping University, 581 83 Linköping, Sweden; valjo@ifm.liu.se (V.J.); mikael@alminica.se (M.S.); rosya@ifm.liu.se (R.Y.); 8 Crystal Growth Lab, Materials Department 6 (i-MEET), Friedrich-Alexander University Erlangen-Nürnberg (FAU), 91058 Erlangen, Germany; Philipp.Schuh@fau.de (P.S.); michael.schoeler@fau.de (M.S.); manuel.kollmuss@fau.de (M.K.)

**Keywords:** 3C-SiC, heteroepitaxy, bulk growth, compliant substrates, defects, stress

## Abstract

In this review paper, several new approaches about the 3C-SiC growth are been presented. In fact, despite the long research activity on 3C-SiC, no devices with good electrical characteristics have been obtained due to the high defect density and high level of stress. To overcome these problems, two different approaches have been used in the last years. From one side, several compliance substrates have been used to try to reduce both the defects and stress, while from another side, the first bulk growth has been performed to try to improve the quality of this material with respect to the heteroepitaxial one. From all these studies, a new understanding of the material defects has been obtained, as well as regarding all the interactions between defects and several growth parameters. This new knowledge will be the basis to solve the main issue of the 3C-SiC growth and reach the goal to obtain a material with low defects and low stress that would allow for realizing devices with extremely interesting characteristics.

## 1. Introduction

Wide band-gap (WBG) semiconductor devices based on both silicon carbide (SiC) and gallium nitride (GaN) can lead a revolution in power electronics through its faster switching speeds, lower losses, and higher blocking voltages [1]. Furthermore, their properties enable higher operating temperatures and increased power densities, but until now the benefits shown by WBG power electronics have not been fully realized due to the high costs of the material and reliability challenges.

Silicon carbide is a material presenting different crystalline structures called polytypes [2]. To date, only the two hexagonal structures 4H and 6H-SiC are commercialized, while the cubic form (3C-SiC) is not used until now in power devices despite the large effort of the last years and several hundreds of papers published for each year. All these polytypes have similar benefits over silicon such as higher breakdown fields (2–4 MV/cm) and the larger energy band-gap (2.3–3.2 eV). The cubic silicon carbide is the only polytype that can be grown on silicon wafers. This approach reduces the cost as no SiC substrate is used and only the silicon carbide layer thickness required for the specific application is grown on a cheaper Si substrate. This technology also offers the potential for faster scale-up with wafer size compared with the development of larger diameter hexagonal SiC wafers. In principle, with a large reactor, a 300 mm Si wafer can be obtained using the present process.

Both 3C-SiC and GaN can work in the same breakdown voltage range (200–1200 V). 3C-SiC is more appropriate for high-current applications due to its high thermal conductivity, while GaN better fits in RF applications because of the high saturated electron velocity. The lower band-gap of 3C-SiC (2.3 eV) in comparison to 4H-SiC (3.28 eV) is often viewed as a negative aspect with respect to other polytypes. The lower band-gap brings a positive effect because the lowering of the conduction band minimum causes a reduced density of states at the SiO_2_/3C-SiC interface [3]. As a consequence, it has been demonstrated that the metal oxide semiconductor field effect transistor (MOSFET) on 3C-SiC has the highest channel mobility (above 300 cm^2^/V/s) ever presented on any SiC polytype [4]. This produces a large reduction in the power consumption of power switching devices [5]. A remaining challenge in both 3C and 4H-SiC is the electrical activity of extended defects. It is identified as the major problem for electronic device functionality. The mechanisms of defect formation must be clarified and the methods for their reduction developed to reach full functionality and high yield [6]. So far, the growth of 3C-SiC on silicon has been demonstrated on 150 mm Si wafers [7,8]. The process is feasible with up-scaled reactors on 200 mm or 300 mm wafers.

Another problem to solve concerns the intrinsic stress created during the growth process due to the lattice mismatch between 3C-SiC (4.36 Å) and Si (5.43 Å) [9]. Thermo-elastic stress also appears at the post-deposition cooling due to the 8% difference in the thermal expansion coefficients between these two materials [10]. This results in stress which induces the formation of various planar or extended defects in 3C-SiC. These defects produce a considerable degradation of the crystalline quality of the epitaxial layer [11].

There are two types of defects in the epitaxial 3C-SiC layers. Anti-phase boundaries (APBs) are planar defects that are formed at the geometrical separation of two 3C-SiC grains. The grain differs by a 90° rotation in the Si(100) growth plane [12]. These are described as anti-phased domains (APDs) that are formed by the steps present on the Si surface. A second defect type is the stacking fault along the {111} planes. Most research work highlights the intrinsic nature of these defects and points out that a reduction of their density is possible by increasing the film thickness. The electrical activity of the extended defects in 3C-SiC is a dominant problem for electronic device performance. Clearly, a reduction of these defects is essential to improve the yield of power electronic devices [13].

In the last years during the CHALLENGE project [14], we have been active in developing two different approaches. In the first one, we have used several structured substrates (compliance substrates) [15,16,17] to reduce the defect density and stress. This approach maintains the attractive low cost of the silicon substrate material. However, it does not take advantage of the high heat dissipation of 3C-SiC because it is limited by the low thermal conductivity of silicon. The second approach of the bulk growth has a higher cost due to the higher cost of the 3C-SiC substrate but allows to take advantage of the high thermal conductivity of 3C-SiC in the devices. Furthermore, there is an advantage in the device processing because of the possibility to use the high-temperature processes already developed in 4H-SiC processing (e.g., epitaxy, ion implantation activation, etc.). The use of Si as a substrate has the main advantage, with respect to 4H-SiC, of reaching large diameters directly without the long and expensive process of the traditional diameter enlargement. In addition, the lower band-gap and higher channel mobility of 3C-SiC can produce MOSFET with a lower R_on_ with respect to both Si and 4H-SiC in the breakdown voltage region between 200 and 1200 V [5]. In previous works [18,19], it was observed that in increasing the grown thickness of the material, the density of SFs can be strongly reduced and this will produce an overall improvement in the electrical performance of the devices.

The bulk growth of hexagonal 6H and 4H silicon carbide polytypes can be considered mature while the same cannot be said about the cubic polytype (3C-SiC) [20,21]. Different approaches such as the modified PVT (Physical Vapour Transport) (M-PVT) or continuous-feed PVT (CF-PVT) method have been presented for bulk growth over the last decade [22,23,24]. Only the methods based on the sublimation sandwich implemented by Tairov et al. [25] could allow for the formation of 3C-SiC [6,26]. A major drawback for all the mentioned bulk growth processes is the lack of available high-quality seeding material. One widely investigated approach makes use of hexagonal SiC wafer materials and explores switching to 3C-SiC. Another promising approach is based on the heteroepitaxial growth of cubic silicon carbide on silicon (3C on Si) using chemical vapor deposition (CVD). This method faces some challenges caused by the lattice mismatch of approximately 20% between silicon and 3C-SiC, and the difference in thermal expansion [27]. The research in this area has advanced in major ways over the last years and has been revitalized to use the material as seed for bulk growth processes [6].

## 2. Materials and Methods

Several different compliance substrates have been realized to improve the quality of the heteroepitaxial growth.

Inverted silicon pyramids (ISP) were fabricated on (001) Si wafers by deep UV lithography. The structures consisted of 700 nm-wide square geometries with a 1.4 μm pitch. A thin layer of stoichiometric silicon nitride deposited by LPCVD with a thin buffer layer of thermal silicon dioxide was used as a hard mask. The layer was etched by a fluorine-based plasma and the silicon substrate was etched by a 45 wt.% KOH @ 70 ^◦^C solution.

In the second compliance substrate, the samples consist of a silicon (0 0 1) substrate with 2 μm of Si_1−x_Ge_x_ grown on top and a cap of 10 nm or 20 nm thick Si. From simulations [28], the ideal value of [Ge] for the lattice match was calculated to be around 12%. In our studies, three germanium concentrations were used at 10%, 12%, and 15% The heteroepitaxial growth of the 3C-SiC films on the ISP and Si-Ge buffer substrates was realized using a chemical vapor deposition process by NOVASiC in a horizontal hot-wall reactor operating with standard silane/propane/hydrogen chemistry [29].

In the third compliance substrate, the Si(111) substrates were patterned into hexagonal arrays of pillars on 100 mm wafers. The standard Bosch process in the shape of hexagonal prisms 8 μm deep and 2 or 5 μm wide that were separated by 2 or 3 μm trenches was applied to fabricate the pillars. The pillars had 100 μm hexagonal patches separated by 5 μm trenches to avoid their contact and reduce substrate bowing. To further improve the pillars’ properties, a three-step procedure was used: (i) a vertical Bosch process was first applied to dig the pillars; (ii) one isotropic dry carving was next applied to create the under etching and necking; and (iii) some oxidation-stripping cycles were used to finally smooth the sidewalls and reduce the top layer thickness of the pillars. The heteroepitaxial growth was realized in a LPE M10 reactor using a trichlorosilane–ethylene hydrogen chemistry [6].

For the bulk growth, 3C-SiC seeds were realized on Si (100) substrates using a chemical-vapor deposition process in a horizontal hot-wall reactor (LPE M-10, Catania, Italy). The silicon and carbon precursors were trichlorosilane (SiHCl_3_ or TCS) and ethylene (C_2_H_4_) using hydrogen (H_2_) as a gas carrier. The process was implemented in a low-pressure regime (100 mbar), wherein the epitaxy started at 3 μm/h, then increased to 6 μm/h, and finally further increased to 30 μm/h. This process resulted in a thick layer of about 70 μm. In the next step, the temperature was increased above the melting point of silicon. This resulted in the Si substrate being fully melted inside the CVD reactor. The remaining freestanding SiC layer was used as a seed layer for the following homoepitaxial growth which used low-pressure regime at different temperatures (between 1600 and 1700 °C). A growth rate of 60 μm/h was used to increase the substrate thickness for two hours [6]. The first 20 microns of the 30 µm layer were highly doped and the last 10 microns were low doped for device realization. Nitrogen was used for n+ and n-type layer formation. The total thickness of the 3C-SiC homoepitaxial samples was about 200 μm (confirmed by SEM analysis).

**Figure 1:**
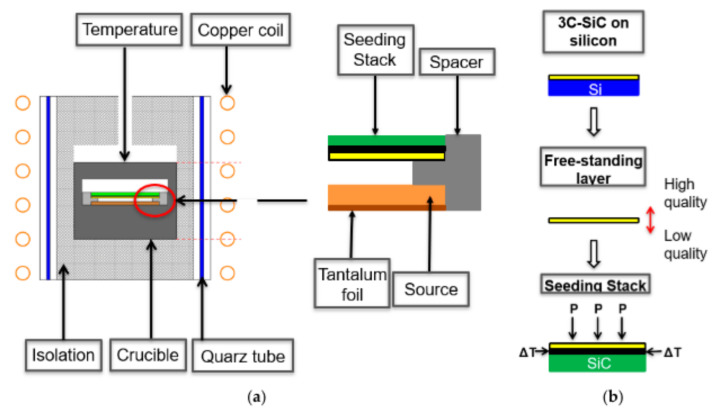
(**a**) PVT reactor used for the sublimation growth and hot zone consisting of a tantalum foil to acquire carbon, the source material, a spacer to separate the source and seed, and the manufactured seeding stack. (**b**) Schematic of the seed manufacturing process. Starting from 3C grown by CVD on a silicon substrate, the silicon is removed by chemical wet etching and subsequently the thin freestanding 3C layer is merged to a polycrystalline SiC carrier for mechanical stabilization and backside protection (see Reference [32]).

Another approach for the bulk growth is to use enhanced sublimation epitaxy (ESE), which is a modified physical vapor transport (PVT) growth technique originating from a patent-protected epitaxial SiC growth method [30] developed by researchers at the Linköping University. A typical ESE growth setup is shown in Figure 1. The exclusiveness of the ESE is the distance between the source and the substrate, as well as the character of the source material itself. In a standard PVT setup, the distance between the source and the substrate is more than 10 mm, while in the ESE it typically varies between 0.5 and 2 mm. Such a distance is sufficient to create a large enough temperature gradient, which is the main driving force for the growth. In addition, compared to PVT, such a short distance allows for the more direct transfer of SiC vapor species from the source to the substrate, with a much lower interaction with the graphite walls. In PVT, the source material is a polycrystalline SiC powder, while in the ESE, the powder is replaced by a polycrystalline SiC plate. Furthermore, tantalum (Ta) foil is inserted into the graphite crucible. At growth temperatures, it reacts with carbon-bearing species and forms TaC. In this way, the vapor-phase composition inside the crucible is enriched with Si, which is beneficial for the enhancement of 3C-SiC stability. The combination of the short distance, Si-enriched growth ambience, and stochiometric monolithic source makes ESE an excellent technique for the growth of high-quality SiC layers in a vacuum (1 × 10^−4^ mbar) at temperatures below 2000 °C. Such growth conditions are favorable to induce SiC conversion from hexagonal to cubic polytypes, which are known to be more stable at temperatures below 2000 °C.

Using a transfer process developed at FAU Erlangen-Nurnberg [31], growth of the bulk 3C-SiC with reasonable dimensions and thicknesses was demonstrated using such seeding material and the approach of closed space PVT (CSPVT), [26,32], in its original concept also known as Sublimation epitaxy (SE), was utilized.

Starting from 3C-on-Si material grown by CVD (LPE M10, Catania, Italy), high-temperature stable seeds for bulk growth in a PVT setup can be fabricated according to Figure 1b. First, the CVD seeds are cut to get the desired dimensions. At the start of the project, a diamond wire saw was used. However, the size of the samples that could be prepared was limited to approximately 12.5 × 12.5 mm^2^, as the sawing process induced cracks along the <110> direction in the 3C layer due to the applied mechanical force [33]. Therefore, a change to a multipulse-laser ablation technique [34,35] was made. As the melting point of silicon (1419 °C) is well below the required temperatures for PVT growth (<1800 °C), a removal of the silicon substrate is necessary. Otherwise, the molten silicon would immediately react to silicon carbide with the graphite crucible and graphite isolations used in PVT setups [24]. Therefore, after the cutting of the samples, a wet-chemical etching of the silicon substrate with HNA (HF: HNO_3_: H_2_O) was performed [36,37], resulting in a thin (typically between 20 and 50 µm) free-standing 3C-SiC layer. The etched layers featured a high-quality growth front as well as a defect-rich backside associated with the former transition area between Si and 3C-SiC. Subsequently, the layers were merged to a polycrystalline SiC-carrier with the high-quality growth front facing up. This step is necessary for the mechanical stabilization of the thin 3C layers and to prevent a backside sublimation during the sublimation growth. For the merging, a carbon glue with the main component of 1-Methoxy-2-propanol acetate [31] was used and both a combined heat and pressure treatment was applied. After the merging, residues of the carbon glue that could remain on top of the growth caused by an overflow of the glue were removed to maintain a high-quality starting point for the sublimation growth [38].

## 3. Results and Discussions

### 3.1. Flat Substrates

The 3C-SiC grown on silicon has still high defectivity even though strategies for the elimination of a large plethora of defects are achieved. Common three-dimensional defects of 3C-SiC grown epitaxially on silicon, such as protrusions, twinned regions, antiphase domains, and polytypes inclusion, are eliminated or strongly reduced. On the contrary, the elimination of dislocation, stacking faults, and stress in the 3C-SiC layer is far from being solved. The hetero-interface is the principal “source” of such defects. Indeed, lattice mismatch among Si and SiC, as well as the disparity in thermal expansion coefficients, induced the formation of stress in the film and the formation of both SFs and dislocations.

**Figure 2:**
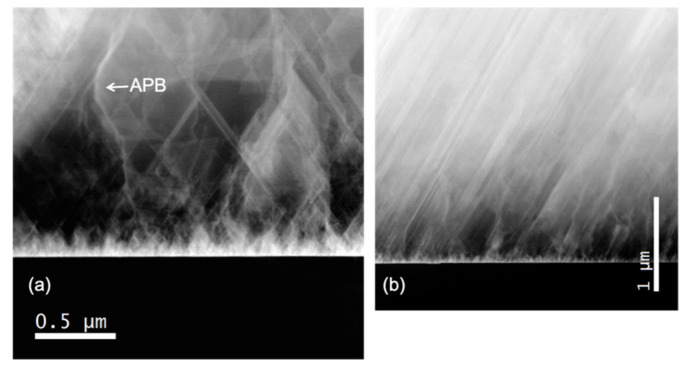
(**a**) TEM cross-section of a 3C-SiC layer grown on on-axis silicon. APBs and SFs in opposite directions are visible. (**b**) TEM cross-section of a 3C-SiC layer grown on off-axis silicon. No APBs can be observed while only SFs in the step direction are visible.

For example, stacking faults in 3C-SiC can come out from Lomer dislocations’ dissociations at the hetero-interface: Lomer dislocation (that forms naturally due to the lattice mismatch at the hetero-interface) with the Burgers vector of a/2 $\left[\bar{11}0\right]$ can dissociate in two partial dislocations, with the Burgers vectors a/6$[\bar{21}1\bar{]}$ and a/6$\left[\bar{12}1\right]$ where a is the lattice constant. The two stacking faults propagate through the epitaxial layer and can approach the surface. A cross-view image of the Si/SiC hetero-interface is shown in Figure 2. In this figure, SFs are the oblique bright lines. A region near the hetero-interface with a high number of SFs is highlighted and the density of SFs decreases, moving away from the hetero-interface. In the “on-axis” substrate (Figure 2a), the SF can intersect and form another kind of defect such as the “Lomer lock”. The formation of this linear extended defect modifies the mechanical properties and more interestingly can avoid the propagation of the SFs in the epilayer. Unlike the case of the “on-axis” substrate on which the SFs can interact with each other in the “off-axis” substrate (Figure 2b), SFs can arrive at the surface because they have the same orientation and are not able to cross each other. Even in this case, a region of high density of SFs is apparent near the hetero-interface.

Another defect that is present only in the “on-axis” image is the anti-phase boundary (APB) or also called “inverted domain boundary” (IDB)) (Figure 2a). This defect is a 2D defect and it is the boundary between two 3C crystals rotated by each other by 180° around the [110] axes. It is observed in Figure 2a as a curved bright line. This defect preferentially lies on the (110) or (111) plane and has a particular atomic structure: it is coherent and can couple with SF in a complex way. In the (110) plane, the structure is made up of a bent Si-C bond that generates a square and a semi-octahedral configuration with unaltered bonds, while in the (111) plane it resembles a twin with a Si–Si bond. In the last case, it can be associated with an SF. For more detail, the reader can refer to [39,40]. The propagation of IDBs within the crystal appears to be extremely complex, resulting in “complex IDBs” interacting with SFs. Moreover, we noticed that IDBs can also end and generate SFs. The presence of “disconnection” (which are steps with a Burgers vector associated in the IDB) might cause such behavior.

The different orientations of the substrates ((100), (111), or (110)) produce a different structure of the material on the surface [41] and a difference in the stress after the deposition.

### 3.2. Compliance Substrates: Pillar Growth

**Figure 3:**
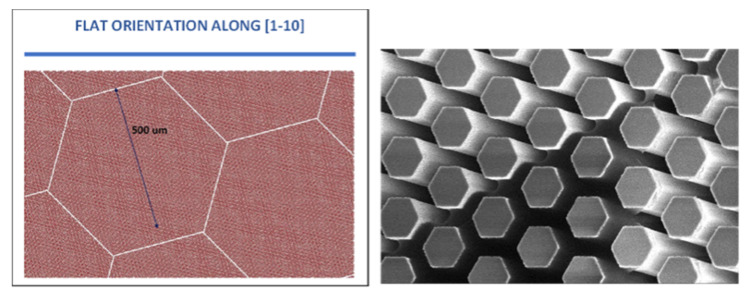
Pattern (left) and shape of the pillars (right). Both pillars and the pattern have a hexagonal structure.

The pillar technology is intended to doctor the thermal strain of the deposited SiC film by growing a suspended (thick) layer on top micrometric Si pillars, which eventually bend to accommodate the larger thermal retraction in the cooling down of the SiC film with respect to the Si substrate. The pillars are patterned in arrays in the Si substrate by a dry etching process [42,43] (Figure 3). The <111> orientation is the most critical one for stress accumulation with film thickness on flat substrates (less than 1 µm without cracks) and also provides a better quality of the deposited material on pillars. Therefore, the shape of the pillars (hexagonal in cross-section) and the arrangement of the arrays (still hexagonal in the pattern), which are suitably rotated with respect to the wafer flat to reduce the slanted 111 facet extension, have been optimized for the <111> growth orientation, wherein the pillar technology could provide the most important contribution.

**Figure 4:**
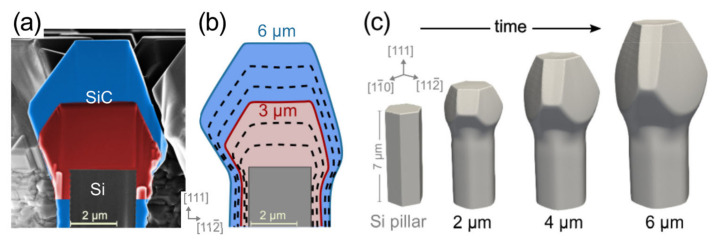
(**a**) SEM (1-10) cross-section view of the upper part of a SiC crystal after 3 µm (red) and 6 µm (blue) deposition on top of a 2 µm-wide Si pillar (gray), which is 8 µm tall. (**b**) Phase-field simulation profiles for the same conditions of (**a**) reproduced every 1 µm deposition. (**c**) 3D view of the evolution sequence obtained from simulations (see Reference [44]).

Understanding and controlling the 3D crystal growth and subsequent coalesce dynamics are the keys to optimizing the patterning and obtaining high-quality 3C-SiC suspended layers on the underlying Si pillars. To this goal, an extensive theory-experiment analysis of the evolution of the crystal growth has been performed and detailed in Reference [44]. First, the faceted growth of the individual SiC crystals has been characterized, as illustrated in Figure 4, by comparing the profiles of samples grown at different times with phase-field simulations based on the kinetic growth model of Reference [15]. The unknown facet-dependent growth rates to be set in the model have been extrapolated by fitting the simulation profiles to the experimental ones, resulting in a good match between Figure 4a and b. Once calibrated, the simulations allow us to investigate all intermediate stages of the growth (c), as well as the subsequent dynamics of merging between neighboring crystals.

**Figure 5:**
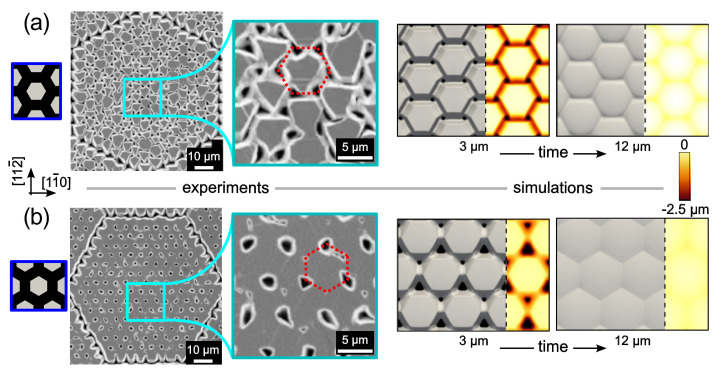
Comparative analysis of the coalescence of SiC crystals grown on Si pillars for the two different patterns with pillar rows along (**a**) the [11-2] and (**b**) [1-10] directions from both experiments and simulations. SEM views are reported for samples obtained after 12 µm SiC deposition on 5 µm large prismatic Si pillars, spaced by 2 µm gaps. The magnified views highlight the different patterns of holes left by partial coalescence. Simulation snapshots are shown for both the 3 and 12 µm deposition. The colored regions show the variations in height by the colormap. A smoother profile is achieved in case (**b**) (see Reference [44]).

As illustrated in Figure 5, two limiting cases have been studied, corresponding to a 90° rotation of the hexagonal pillar pattern. In case (a), pillar rows are along the [11-2] directions so that coalescence occurs with a six-fold symmetry by bridging the large {111}-C terminated facets with the smaller {100} ones, leaving six identical holes to fill at the latest stages. In the same way, in case (b), pillar rows are aligned along the [1-10] direction such that coalescence occurs at facet edges, resulting in a three-fold symmetry arrangement with a larger hole in between {111}-C facets and smaller ones at the crossing of {100} facets. As made evident by simulations, this latter arrangement is the most convenient, returning a smoother surface profile after the deposition of about 12 µm.

**Figure 6:**
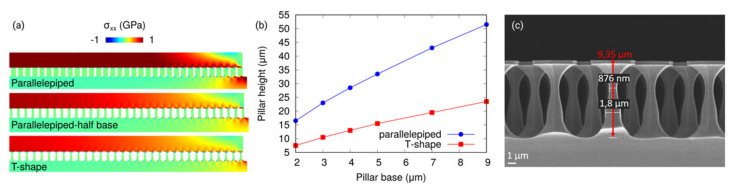
(**a**) Color maps of the xx component of the stress tensor (σ_xx_) for three 3C-SiC epilayers grown on array of pillars with different geometries. Top: parallelepiped pillars, spaced by 2 µm and with a base width of 5 µm. Center: parallelepiped pillars, spaced by 4.5 µm and with a base width of 2.5 µm. Bottom: T-shape pillars, spaced by 2 µm and with a maximum base width of 5 µm. (**b**) Plot of the height of the pillars as a function of the width of the pillars that is needed to guarantee a curvature radius of the sample that is larger than 10 m (acceptable for post-processing of 4′ wafers). A (111) Si substrate is considered. (**c**) SEM image of the T-shape pillars (adapted from Reference [46]).

The strain relaxation in the 3C-SiC epilayer is enabled by the tilting of the pillars underneath. As reported in Reference [45] for the case of Ge grown on Si pillars, the deformation can be described as a rigid-body rotation of each pillar. It is possible to conclude that the capability to rotate strongly depends on the aspect ratio of prismatic (or paralepidid) pillars. Indeed, as shown in Figure 6a for the pillar at the periphery of the array exhibiting the maximum deformation, the rotation mechanisms and consequently the stress relaxation are larger for a smaller pillar width. Another important parameter that controls the relaxation in the 3C-SiC epilayer is the height of the pillars. Indeed, as observed in Figure 6b for different patch sizes, the higher the pillar, the better is the strain relaxation. The stress (and strain) relaxation at the center of the array decreases when the patch size is increased, at a fixed pillar aspect ratio, asymptotically matching the reference case without any pillar when the patch size tends to be infinite. The relaxation of the elastic energy in the epilayer can be enhanced also by changing the pillar spacing or, more importantly, the pillar shape. Indeed, if compared to the standard parallelepiped pillars, T-shaped ones (Figure 6c) offer a higher capability to rotate, being thinner in the intermediate section of the pillar. This results in a lower residual stress or equivalent strain, as shown in Figure 6a. The T-shape (Figure 6c) case is comparable to the one with parallelepiped pillars, characterized by a base of half-size and a larger pillar spacing, with the advantage that the T-shape ones have a larger top surface for each pillar, above which the SiC can be grown. According to the approach discussed in Reference [46], in Figure 6b, the relation between the width and height of the pillars is plotted to guarantee a curvature radius of the sample that is larger than 10 m. The curvature radius is calculated from the average residual strain in the epilayer according to the Timoshenko formula for planar bilayers [47].

### 3.3. Compliance Substrates: SiGe Buffer Layer

Another attempt to grow a high-quality 3C-SiC epilayer on a silicon substrate was done by introducing a buffer epitaxial layer of Si_1−x_Ge_x_ between Si and the SiC. We choose a layer of Si-Ge because Si and Ge have the same FCC structure and are perfectly miscible: the stoichiometry and lattice parameter can be decided a priori. This fact has important implications: fine-tuning the lattice parameter in such a way to minimize the mismatch due to the 4/5 ratio among the Si and SiC atomic layers is possible. Indeed, as already reported, 3C-SiC and Si show roughly a 20% lattice mismatch, implying that four layers of Si almost “equate” to five layers of SiC (the 4/5 rule). The extra plane of SiC creates Lomer and misfit dislocations, thus stacking faults. Nevertheless, the 4/5 rule is not exact and a mismatch (dependent on temperature) also exists between four layers of Si and five of SiC. This mismatch creates stress with the formation of extra dislocations and stacking faults. The adoption of a buffer layer of Si-Ge can also reduce the thermal stress due to the thermal expansion coefficient mismatch (between Si and SiC) caused by the cooling of the sample from the growth temperature (about 1400 °C) to room temperature.

**Figure 7:**
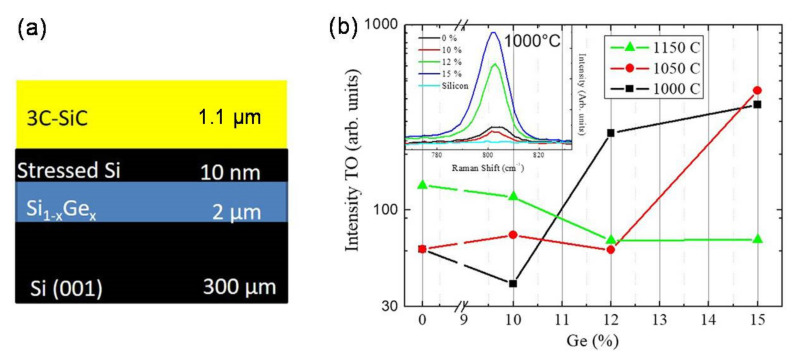
(**a**) Schematic of the sample structure. The image is not to scale. (**b**) 3C-SiC TO peak height with respect to nominal Ge concentration for several carbonization temperatures. The spectra of samples that have undergone 1000 °C carbonization are shown in the inset (adapted from Reference [16]).

In Figure 7, we show the structure of the sample used in the experiment: 10 nm Si cap on 2 μm Si_1−x_Ge_x_ grown over a 300 μm silicon (0 0 1) substrate. The thin Si capping layer thickness is lower than the “critical thickness” to avoid the formation of interfacial defects at the Si/Si_1−x_Ge_x_ interface and it was introduced as a seed for the carbonization step. The intensity of the transverse optical (TO) peak of SiC at 796 cm^−1^ is shown in Figure 7b for different carbonization temperatures and buffer layer compositions. The TO Raman peak is forbidden for perfect 3C-SiC grown on (0 0 1) substrates (due to the selection rules) and its presence is associated with twins and poly-crystals. It was observed that greater Ge content and lower temperatures (1000 °C, 15%; 1050 °C, 15%; and 1000 °C, 12%) lead to a high TO mode intensity. In these samples, we find Ge segregation at the interface between the SiC and SiGe layer. Ge segregation implies the formation of poly-crystals, while samples with lower concentrations and higher carbonization temperatures lead to a mirror-like surface morphology and a lower value of TO intensity indicates a higher SiC quality.

We also demonstrated that the carbonization temperature and composition of the layer control the quality of the SiC film. It is also possible to achieve a higher quality with respect to film grown on virgin silicon [16].

### 3.4. Compliance Substrates: Inverted Silicon Pyramids (ISP)

One of the most interesting attempts to grow a high-quality 3C-SiC epilayer on a silicon substrate was done by creating a structured substrate. The structure came from the following consideration: the SFs lie on {111} planes and can interact with each other, stopping the propagation. Consider two SFs laying, for example, in the (111) and (11-1) planes; they can cross and the structure is able to stop the propagation of one or even both SFs. This clearly improves the crystalline quality of the film surface because the SFs remain buried in the epilayer. The rate of SF annihilation is inversely related to SF density, however, by means of the inverted silicon pyramid (ISP) compliant substrate, allowing for a significant drop in SF concentration just within a few microns.

Its unique shape can concentrate SFs in tiny areas, enhancing the phenomenon of SF annihilation [48].

**Figure 8:**
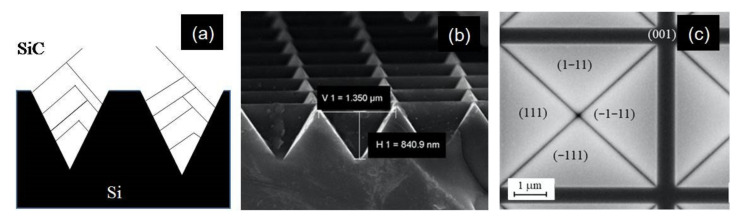
(**a**) Schematic cross-section view of the effect of the ISP compliant substrate on the SFs. Silicon and silicon carbide are drawn as black and white regions. Blue lines are SFs. (**b**) Cross-view SEM image of the ISP structure. (**c**) Plane-view SEM image. The four (111) planes of the pyramid are shown, as well as the (001) region among the two pyramids (adapted from Reference [17]).

In Figure 8a, we show a schematic cross-section view of the effect of this compliant substrate. Silicon and silicon carbide are drawn as black and white regions. Blue lines are SFs which either generate an X-shaped defect known as the forest dislocation or self-annihilate, resulting in a system known as the Lomer lock, or end on an existing SF, producing a so-called ”λ-shaped” defect. In Figure 8b,c, SEM images of the ISP structure are shown in plane cross-view and plane-view. The four (111) planes of the pyramid are shown, as well as the (001) zone among the two pyramids.

The drawback of the use of this substrate is the formation of APB due to the different polarities of the (111) faces of the SiC [17]. Nevertheless, it is well known that the grain boundary density can be greatly decreased through the enhancement of the film thickness.

**Figure 9:**
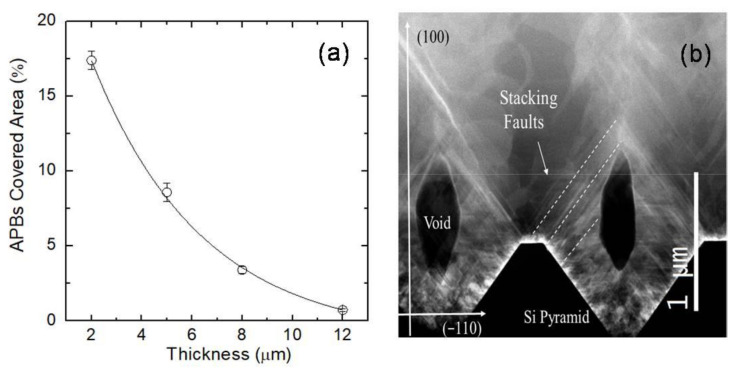
(**a**) Anti-phase boundaries (APBs) covered-area percentages for different thicknesses of the epitaxial growth. (**b**) Cross-section SEM image of 12 um-thick epitaxial 3C-SiC layer grown on ISP (adapted from Reference [17]).

The APBs coverage with respect to layer thickness is depicted in Figure 9a. Despite the fact that the substrate design yields APBs, their concentration was rapidly reduced. Some tenth of microns of the SiC layer is enough to largely reduce the density of APBs. The ISP morphology also induces the formation of buried voids in the epilayer because the (111) face has a slower growth rate than the (100) face. These voids are observed in Figure 9b in which a cross-view TEM image of the 12 μm-thick epitaxial 3C-SiC layer is shown. The generation of voids may be advantageous in reducing the defectiveness of the epilayer. Voids can annihilate SFs and reduce the residual stress in the layer. SFs that cross the void are not able to propagate into the epilayer, reducing the defectivity. It is also feasible to manage the void height by adjusting the growth rate and conditions. In such a way, it is possible to modulate the concentration of SFs arriving on the surface [17].

### 3.5. Compliance Substrates: 4H and 6H-SiC

Hexagonal SiC (4H- or 6H-SiC) is a very promising substrate for the heteroepitaxial growth of 3C-SiC due to the excellent chemical compatibility, thermal expansion, and lattice constant matching. Moreover, contrary to silicon, the hexagonal SiC can be used in high (>1800 °C) temperature processes such as the ESE, the concept of which has been proven to be advantageous for the growth of homo and heteroepitaxial SiC layers at growth rates of up to 1 mm/h [49]. A majority of hexagonal SiC substrates available on the market today can be categorized into off-axis (usually 4 degrees off-oriented towards <11–20> direction) and nominally on-axis substrates. The latter have been commonly used to grow 3C-SiC by sublimation techniques. However, due to difficulties in controlling the spontaneous nucleation of the 3C-SiC island on such substrates and the formation of structural defects called double positioning boundaries (DPBs), it has been challenging to grow 3C-SiC with high crystalline quality. In contrast, the off-axis substrates have been mainly used for homoepitaxy or the bulk growth of hexagonal SiC crystals. The difference between the off-axis and nominally on-axis surfaces is the density of steps. The higher density of steps on off-oriented surfaces enhances the reproducibility of the substrate polytype and significantly reduces the possibility of 2D formation of 3C-SiC on step terraces. Therefore, generally, they have not been considered for the heteroepitaxial growth of 3C-SiC layers. However, it has been demonstrated that under certain growth conditions, excellent polytype stability and a high quality of 3C-SiC can be obtained on research size (7 × 7 mm^2^) 4 degrees off-oriented hexagonal SiC (0001) substrates [22,23].

### 3.6. Hetero-Epitaxy Process: Carbonization

The heteroepitaxy of 3C-SiC on Si is a complex process that is realized in several steps. After the introduction in the reaction chamber, the first step is the etching of the silicon substrate in a hydrogen flux to remove the native oxide (step 1). Then, the second step is the substrate carbonization wherein a flux of the carbon precursor and carrier gas (hydrogen) is introduced in the chamber at temperatures between 900 and 1200 °C (step 2). Subsequently, the temperature should be increased to grow the 3C-SiC layer at temperatures close to the melting point of silicon (step 3). Finally, the temperature is decreased to room temperature (step 4). All these steps have a considerable impact on the quality of the epitaxial layer.

The most critical in the 3C-SiC/Si growth seems to be the second step: the carbonization of the Si substrate. This process is sometimes referred to as “reactive CVD” (R-CVD) because one of the components of the compound (in this case, silicon) is not supplied from the vapor phase but comes directly from the Si substrate that reacts with the gas (“vapor”) species. As a result of carbonization, a thin seed of a few nanometers is formed for the subsequent CVD epitaxy process; it is sometimes denoted as the “carbonization buffer”. The characteristics of this seeding layer are fundamental for the crystalline quality and the stress of the film. From previous studies, it has been observed that for given growth conditions, the morphology and thickness of the carbonization buffer depend strongly on the substrate orientation [50]. Consequently, the conditions to obtain an optimal buffer differ between orientations. For any orientation, the maximal thickness is conditioned by the nucleation density (proportional to carbon supply) and the ratio between the vertical and lateral growth rates (controlled by process pressure and temperature).

During carbonization, initial nucleation centers extend progressively, laterally, and vertically into three-dimensional 3C-SiC islands. Their temperature-dependent growth rate is proportional to the carbon flow rate but remains limited by the Si supply from the substrate; high in the initial stage when surface coverage with 3C-SiC is low, it reduces progressively to zero as the 3C-SiC islands extend, coalesce, and block the Si supply. It is important to mention that the R-CVD growth mechanism remains active until a complete coalescence of the 3C-SiC buffer is achieved, which is sometimes a long process. Consequently, in many cases, the CVD mechanism coexists (intentionally or not) with R-CVD during the initial part of step 3 of the heteroepitaxial growth.

The roughness of the carbonization layer has a large effect on the stress of the entire film: high roughness makes the relaxation of intrinsic stress during the growth easier [51]. The main process parameters that influence the roughness of the carbonization buffer are the temperature of the carbon precursor introduction and temperature of the carbonization plateau.

**Figure 10:**
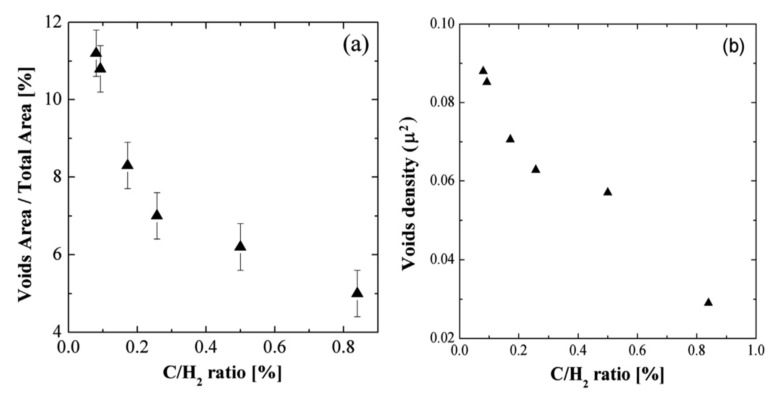
The percentage of void areas occupied with respect to the total observed area (**a**) and voids’ density (**b**) are reported as a function of the C/H_2_ ratio [%] (see Reference [27]).

One of the problems related to the carbonization step is the formation of voids (also called “etch pits”) in the near-interface region of the silicon substrate. These micrometric cavities form from the coalescence of silicon vacancies created in the Si substrate as a consequence of the R-CVD growth mechanism. The major part of voids does not affect the quality of the 3C-SiC film (their presence is sometimes considered as a stress-relaxing factor), although some of them can be at the origin of surface defects in the epitaxial film. Consequently, the void formation should be reduced. This can be achieved through carbonization under a high C/H_2_ ratio, which increases the nucleation density and favors fast-film coalescence that stops the formation of voids. In Figure 10a, the fraction in percentage of the void area with respect to the total observed area as a function of the C/H_2_ ratio are reported. In the same figure, the density of the void as a function of the C/H_2_ ratio is also reported. The effective void areas decreases from 11% to 5% while increasing the flux of carbon atoms. The reduction can be further enhanced by introducing the silicon precursor during the thermal ramp between the carbonization plateau (step 2) and epitaxy (step 3) to form a transition layer. Such intentional mixing of R-CVD and CVD mechanisms further improves the quality of the interface between the 3C-SiC film and Si substrate [27]. In the same paper, it has been reported that the increase of the C/H_2_ ratio also produces an increase in the density of the layer, as well as an increase in the carbonized thickness.

The growth on compliance substrates may require a modification of the carbonization step in order to fit particular substrate-related requirements. This is, for instance, the case for substrates with the Si-Ge buffer for which the carbonization temperature was reduced to below 1000 °C and H_2_ etching (step 1) was excluded from the process in order to preserve a thin Si cap (10 nm or 20 nm), necessary for correct carbonization. In addition, the temperature of the CVD growth (step 3) was lowered to avoid Si-Ge melting. It is important to underline that such a “low temperature” process resulted in a higher quality of the epilayer on Si-Ge with respect to the film grown on bare silicon.

An alternative approach to the formation of the 3C-SiC seed on the Si substrate was recently proposed. Silicon substrates are “pre-carbonized”, meaning that a few nm-thick amorphous carbon (a-C) film is deposited using the plasma immersion ion implantation (PIII) technique. During H_2_ annealing (step 1), carbon reacts with silicon to form oriented 3C-SiC seeds through a solid-state epitaxy mechanism. The standard R-CVD carbonization step is no longer necessary. CVD deposition on such seeds gave satisfactory results on all studied orientations: (100), (110), (111), and (112) [52].

### 3.7. Effect of Growth Rate: Defects and Stress

The growth rate has a large effect on the quality of the 3C-SiC both in terms of its structural quality and stress. It has been observed in a previous paper [53] that the growth rate has a large effect on the density of twins. In fact, in decreasing the growth rate from 10 μm/h to 1 μm/h, a decrease of the twin density of almost a factor of 6 can be observed. A similar (but weaker) effect has been observed also on the rocking curve, which is more sensitive to SFs and point defects [6]. The Full Width at Half Maximum (FWHM) of the rocking curve is reduced both by reducing the growth rate and increasing the thickness.

With decreasing growth temperature, the growth rate has to be reduced or otherwise the deposition may become polycrystalline. This is particularly the case for 3C-SiC growth on the Si-Ge buffer and constitutes a potential limitation for further development of this approach.

For the 3C-SiC growth on ISP substrates, we demonstrated that the height of the void created above the vertex of the pyramid (Figure 9b) increased at higher growth rates. Consequently, the initial stage of 3C-SiC growth on ISP substrates, until reaching complete coalescence, should be performed at a low growth rate.

The growth rate has also an influence on the final stress of the 3C-SiC epilayer. Indeed, as demonstrated in [54], during the growth of the 3C-SiC layer, the intrinsic stress in the layer is continuously relaxing. For a given film thickness, by tuning the growth rate, we can adjust the duration of the relaxation (by the duration of the growth), controlling the final stress of the sample.

### 3.8. Defects in 3C-SiC: SFs and APBs

In 3C-SiC, the most important defects that hinder its use in the microelectronic industry are related to SFs and dislocations. Stacking faults (SFs) are the most important ones dominating over the entire 3C-SiC layer thickness. In literature, three types of SFs are observed depending on the number of atomic planes with the wrong orientation: SFs can have 1, 2, or 3 errors in the stacking sequence and they are called intrinsic (or SF<1>), extrinsic (or SF<2>), or conservative (or SF<3>) [40,55,56].

**Figure 11:**
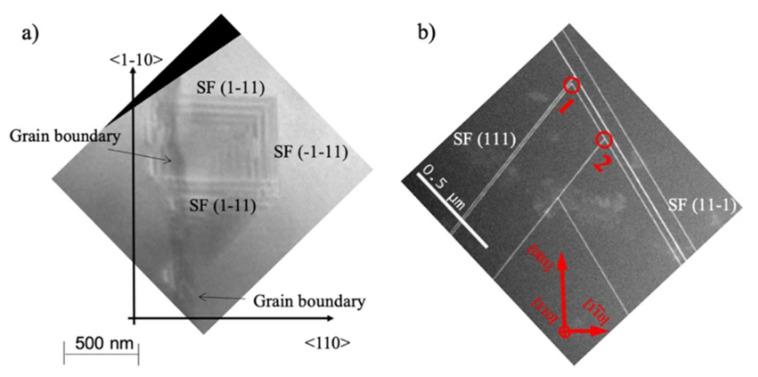
(**a**) TEM image in in-plane view shows four stacking faults that are generated from a grain boundary. (**b**) TEM image in cross-view showing the annihilation of the SF with two different structures (adapted from References [40,60]).

In 3C-SiC, mechanisms for SFs’ self-annihilation exist but there is also the possibility for SFs to be generated [6,40,57,58]. The concomitant presence of these mechanisms leads to the fact that SFs in 3C-SiC can be hardly reduced. The minimum SFs’ densities achieved so far amount to about 10^4^ cm^−1^ in thin films [58]. In Figure 11a, a TEM image in in-plane view shows four stacking faults that are generated from a grain boundary, while in Figure 11b, a TEM image in cross-view shows the annihilation of the SF. In Figure 11a, a vertical grain boundary generates three clearly visible SFs lying in ($1\bar{1}1$), ($11\bar{1}$), and ($\bar{11}1$) planes. Interestingly, two SFs in the ($1\bar{1}1$) planes limit the SF in the ($\bar{11}1$) plane; they are limited on the other side by the grain boundary. The intersection between the SFs ($1\bar{1}1$) and ($\bar{11}1$) is the Lomer−Cotrell partial dislocation and has a Burgers vector of a/6[011 The place in which the grain boundary intersects the SF ($\bar{11}1$) is the place in which the SF ($\bar{11}1$) is generated. In Figure 11b, the crossing of several SFs is shown. This image proves that there are two possible intersections of the SF lying in the (111) and ($11\bar{1}$) planes. The intersection indicated as “1” has an inverted V-shape typical for the formation of a Lomer–Cottrel dislocation. This dislocation, as already reported, has a Burgers vector (a/6[110]) lower than the usual partial dislocation Burger vector (a/6[112]) that borders the stacking fault. The intersection called “2” has a lambda shape; it forms for kinetical reasons. These two configurations can decrease the amount of SF approaching the surface and improve the quality of the epitaxial film.

**Figure 12:**
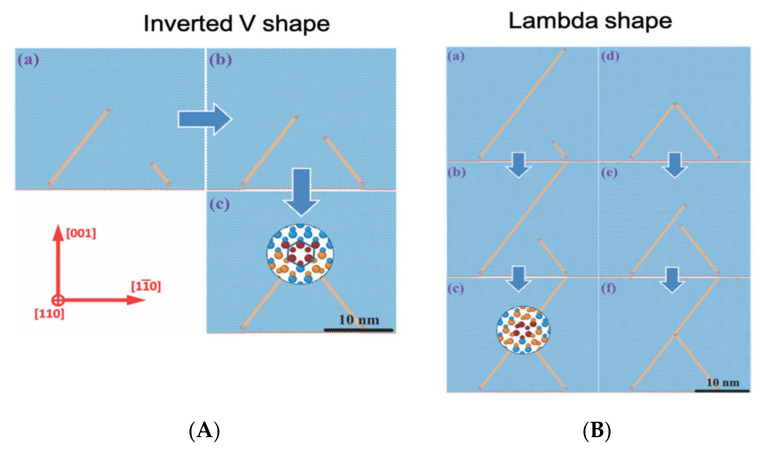
(**A**) Molecular dynamics simulation snapshots of the inverted V-shape configuration.(a–c) The simulation time: (a)—0, (b)—120 ps, and (c)—180 ps. Blue atoms correspond to the Si and C atoms in the cubic diamond lattice, orange atoms belong to the stacking faults. Inset in panel (c) shows the atomic configuration of the formed Lomer–Cottrell lock dislocation. (**B**) Molecular dynamics simulation snapshots of the lambda-shape configuration. in the case of the large distance between the 30° leading dislocations (a–c) and as a result of the interaction of closely spaced 30° dislocations with equal screw components of Burgers vectors (d– f). Simulation time: (a)—0, (b)—360 ps, (c)—540 ps, (d)—0, (e)—60 ps, (f)—200 ps. Inset in panel (c) shows the atomic configuration of the intersection of 30° partial dislocation with crossing stacking fault, also corresponding to the intersection in panel (f). (adapted from Reference [59]).

The annihilation mechanism is considered in more detail in Reference [59]. It is found that the key parameter for the formation of a lambda-shape or an inverted V-shape is the distance between the PDs and the mutual orientation of their Burgers vectors. In the case in which the PDs have Burgers vectors that sum in such a way that the resulting Burger vector is shorter than the initial ones, the partial dislocation attracts to each other and if they are closer by less than about 15 nm, the propagation of both SFs is suppressed with the formation of a Lomer–Cottrell lock. In the case in which the two PDs are far more than 15 nm, they do not interact with each other and can form a Lambda-shape structure. The Lambda-shape can form even if the partial dislocations are close enough, but they repulse. In Figure 12 on the left, a sequence of MD simulation snapshots of the formation of “inverted V”-shaped intersection of stacking faults have been shown. In Figure 12 on the right, MD simulation snapshots of the formation of “λ”-shaped intersections of stacking faults in the case of a large distance between partial dislocations (a–c) and repulsing dislocations (d–f) have been reported. Blue atoms correspond to the Si and C atoms in the cubic diamond lattice, while orange atoms belong to the stacking faults.

**Figure 13:**
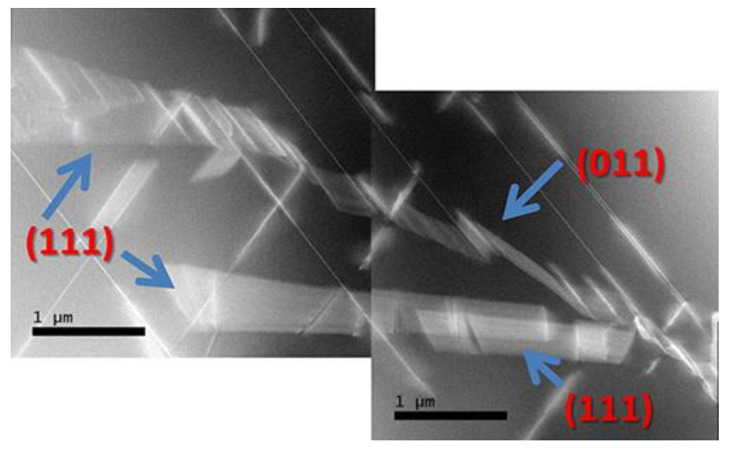
Sequence of STEM (110) cross-view images showing an IDB and its interaction with SFs. The lying planes of IDB and SFs are indicated (adapted from Reference [40]).

The SFs can interact also with other extended defects, such as the inverted domain boundary (IDB) (sometimes called the anti-phase boundary, APB). In 3C-SiC grown on (100) “on-axis” silicon, due to the symmetries of the Si lattice, two equivalent dispositions of the SiC crystal are possible. The two possible orientations are rotated 90° around [001] and, due to the SiC symmetries, a rotation of 90° is equivalent to flip the crystal upside-down. The boundary between two such domains is called IDB (or APB). The SFs can interact strongly with this kind of extended defect of SiC. In Figure 13, a sequence of STEM images showing an IDB interacting with SFs is shown. The image is the projection of the TEM lamellae in the (110) plane. The SFs are observed as straight lines, while the IDB has different lying planes and appears as a ribbon. A close inspection of these images shows that SFs can be generated and annihilated by the IDB: several SFs can be recognized in the figure and some of these are apparent only in the crystal below the boundary, while some others are apparent only in the crystal above the boundary. SFs that are in the lower crystal are not allowed to propagate in the top crystal and in this case, we observe an annihilation of the SF due to the presence of IDB. On the contrary, SFs that belong to the top crystal and are not present in the lower crystal are generated in the IDB. The SFs can be generated during the growth due to interface instability that creates seeds for nucleation; after the nucleation, it expands following the growth of the surface. Eventually, it can collide on an IDB and be annihilated [40]. SFs can be also generated during the cooling down of the temperature after the growth; indeed, temperature gradients can induce stress in the layer. Above critical shear stress, it is known that the formation of dislocations and, in 3C-SiC, the formation of partial dislocation is a thermodynamically favored process.

**Figure 14:**
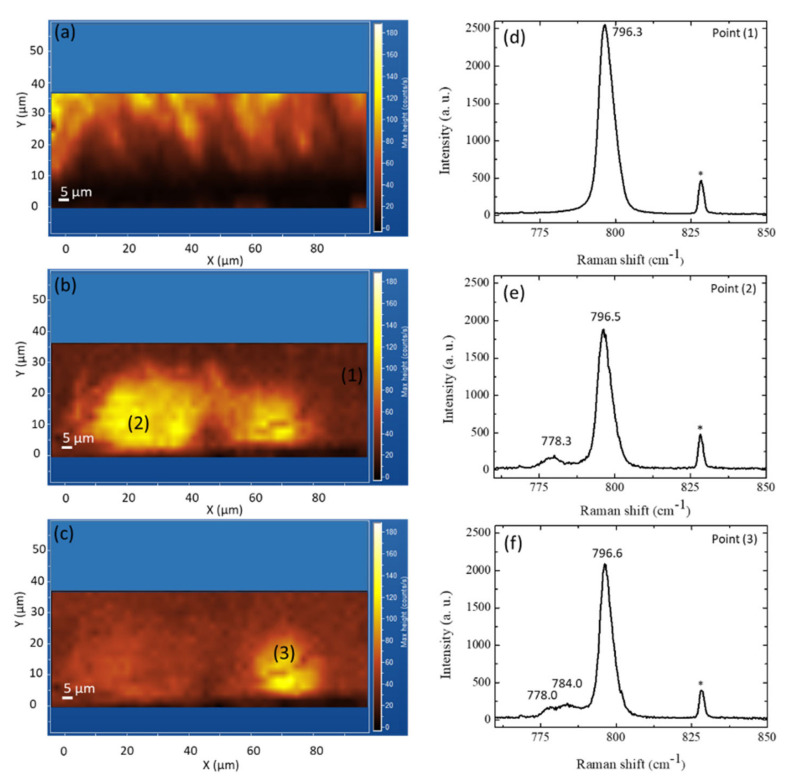
Micro-PL mapping (**a**) at 540 nm and micro-Raman mapping of a 3C-SiC cross-section located at (**b**) 778 cm^−1^ and (**c**) 784 cm^−1^.The interface with the removed Si substrate is shown by point 0 on the Y-axis. Average Raman spectra achieved in the (**d**) area (1) of the map (**b**), (**e**) zone (2) of the map (**b**), and (**f**) zone (3) of the map (**b**,**c**). The laser probe created the peak located at 828.37 cm^−1^ (*) (see Reference [61]).

As previously discussed at the beginning of Section 3.7, different kinds of SFs are present in 3C-SiC. These different types of SFs can be seen as inclusions of different hexagonal polytypes in the cubic structure. In particular, the intrinsic SF can be called a 2H-like SF, the extrinsic one can be seen as a 4H-like SF, and the conservative one can be seen as a 6H-like SF. These different SFs have different energies [60] and different behaviors of these defects should be expected. The room temperature μ-PL map at 540 nm, taken on a 3C-SiC sample in cross-section, is shown in Figure 14a [61]. Moving from the Si-SiC interface towards the top (from 0 μm to 35 μm), the band-edge peak intensity rises, showing a considerable improvement of the crystalline quality, increasing the growth thickness. Figure 14b,c exhibits μ-Raman maps obtained in the same location and indicate certain areas as well as the 3C-SiC/Si interface with greater signal magnitude at 778 cm^−1^ and 784 cm^−1^, respectively. Figure 14d–f displays the mean Raman spectra obtained in areas (1), (2), and (3), which reveal the 3C-SiC TO mode centered at about 796.5 ± 0.2 cm^−1^. Conversely, the mean Raman spectrum obtained in points (2) and (3) reveal an extra peak at 778.3 cm^−1^, as well as two more peaks correspondingly at 778.0 cm^−1^ and 784.0 cm^−1^. These additional peaks can be related to the presence of extrinsic (4H-like and 6H-like) stacking faults. From these data, we can observe that, despite the low energy of the 6H-like SF, it appears that this kind of stacking fault can be observed in larger regions and closer to the surface with respect to the 4H-like SF. More investigations should be done concerning this aspect but we suspect that this large presence of 6H-like SFs could be due to kinetic reasons more than energetic ones. In fact, from the energetic point of view, this SF has the lowest formation energy.

### 3.9. Defects in 3C-SiC: Kinetic Monte Carlo Super Lattice Simulations

The study of the kinetic evolution of a defective system is a difficult task as it requires both atomistic accuracies typical for the molecular dynamics approach and large space-time scales typical for the experimental systems. Within the CHALLENGE project, we developed an ab-initio calibrated kinetic Monte Carlo super lattice (KMCsL) code, [62] offering a good compromise between accuracy and efficiency, which can simulate the results of the growth processes in non-polar SiC as a function of the growth parameters also in terms of defectivity and surface morphologies. Hence, the KMCsL simulations allowed for the investigation of the formation and development of extended as well as point defects over a realistic growth [62]. As an example of a simulation application strongly relevant to the experimental studies, we considered the evolution of anti-phase boundaries (APBs) in 3C-SiC and their interaction with stacking faults (SFs), which is discussed in detail in Reference [57].

**Figure 15:**
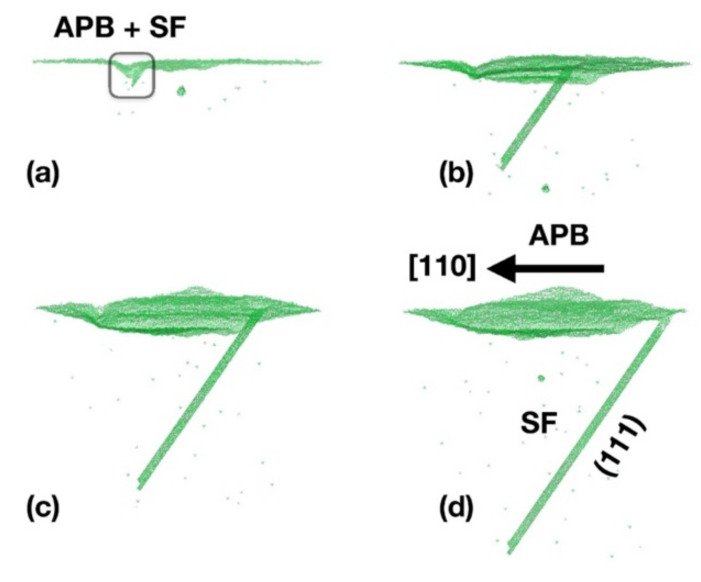

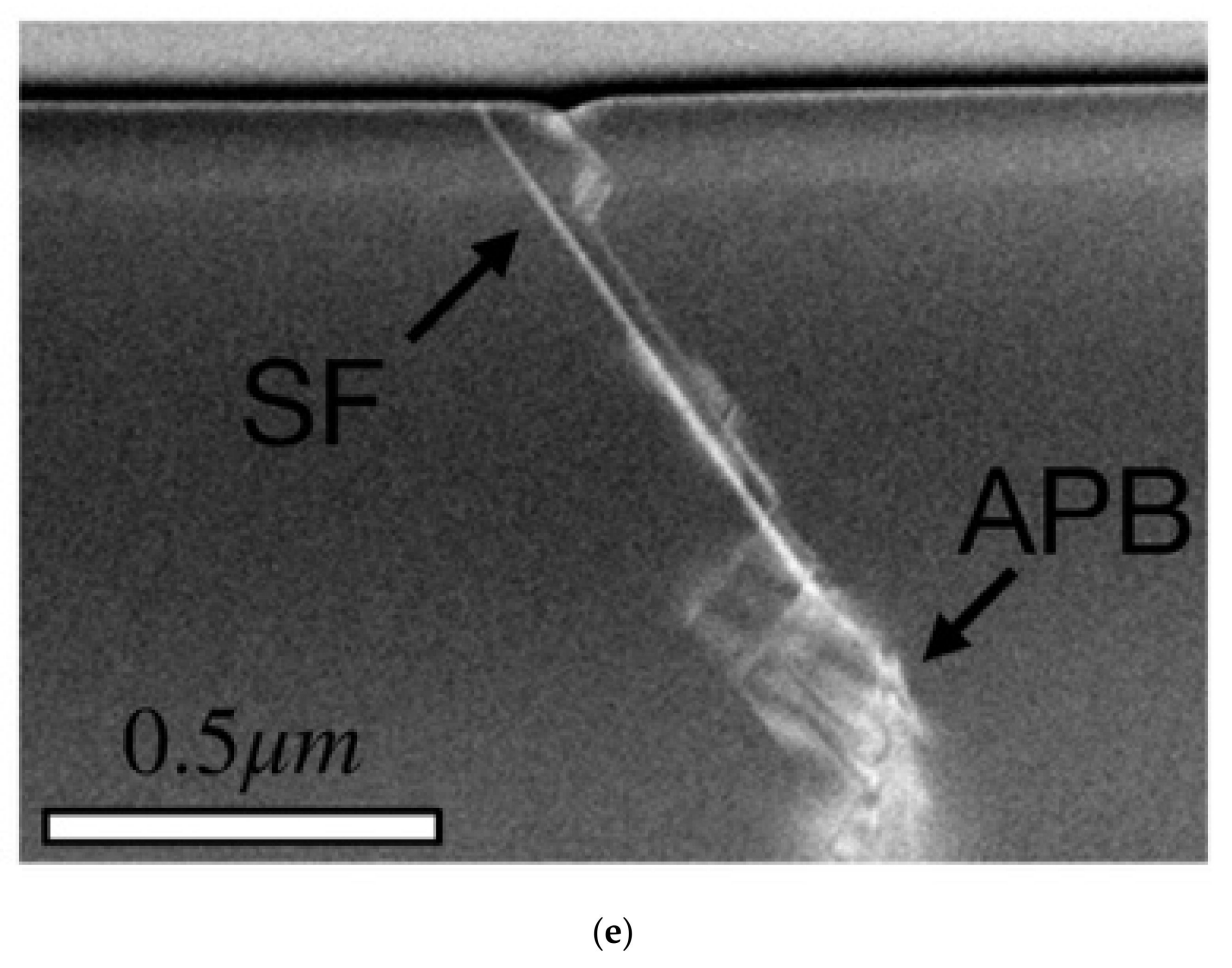
A pair of triple SFs are generated as a result of the surface depletion caused by an APB during 3C-SiC epitaxy along the [001] z-direction. Under-coordinated atoms from several KMC moments: (**a**) triple SFs created by an APB; (**b**–**d**) three consecutive images illustrating the autonomous kinetics of the APB traveling towards the [110] axis; and two formed triple SFs expanding along the (111) planes. (**e**) TEM picture of an SF caused by an APB along the epitaxial growth (001) of a 3C-SiC. It expands on the {111} planes autonomously from the APB kinetics. Moreover, the surface depletion is evident at the (001) surface (adapted from Reference [57]).

Due to the comparable energetics of polytypes, SFs are a frequent and wide-spread extended defect in SiC, with respect to the polytype. They are classified as incorrect atoms sequences in comparison with theoretical polytype stacking arrangements. In the purely hexagonal close-packed (hcp) representation, the polytype sequence is defined as a repeating series of layers made up of Si-C dimers aligned on the hexagonal axis. Dimers in every layer take one of three extremely symmetric locations (often denoted as A, B, and C). If the recurring arrangement is ABC ABC ABC…, etc., extending along the crystal <111> axes, the cubic 3C-SiC (zinc-blend) configuration is produced. As a result, the existence of {111} planes on the surface depletion allows for an increase in SF production. The formation of a pair of triple SFs (SF <3>: three bilayers not in the correct crystalline structure) from the surface depletion caused by an APB is shown in Figure 15. A series of pictures of the under-coordinated atoms obtained at various KMC intervals are displayed. The existence of three-fold coordinated Monte Carlo particles at its border leads to the generation of the triple SF in this depiction (i.e., at the corresponding partial dislocation). We note that the stacking sequence of the triple SF (sometimes termed micro-twin, i.e., ABC ABC ACB ABC ABC ABC) divides two crystal areas in pristine epitaxial order. The only atoms out of the right crystal locations are those within the extended defect. The APBs’ localized asymmetry as well as the existence of the {111} faceted surface result in the development of a triple SF (due to polytype instability) (see Figure 15a,b). When the APB generates the SF, the two extended defects (SF and APB) maintain separate kinetics: the APB proceeds to move through the [110] plane, while the SF expands on the (111) plane (snapshots b, c, and d of Figure 15). A TEM picture of an SF generated by an APB along the epitaxial growth of a 3C-SiC (001) substrate is shown in Figure 15e. It expands autonomously from the APB kinetics along the {111} planes. Moreover, surface depletion can be seen in the correspondence of the (001) surface. These composed structures of proximal APB and SF-type defects have been also evidenced by the conductance maps in this paper.

### 3.10. Defects in 3C-SiC: Electrical Effects

**Figure 16:**
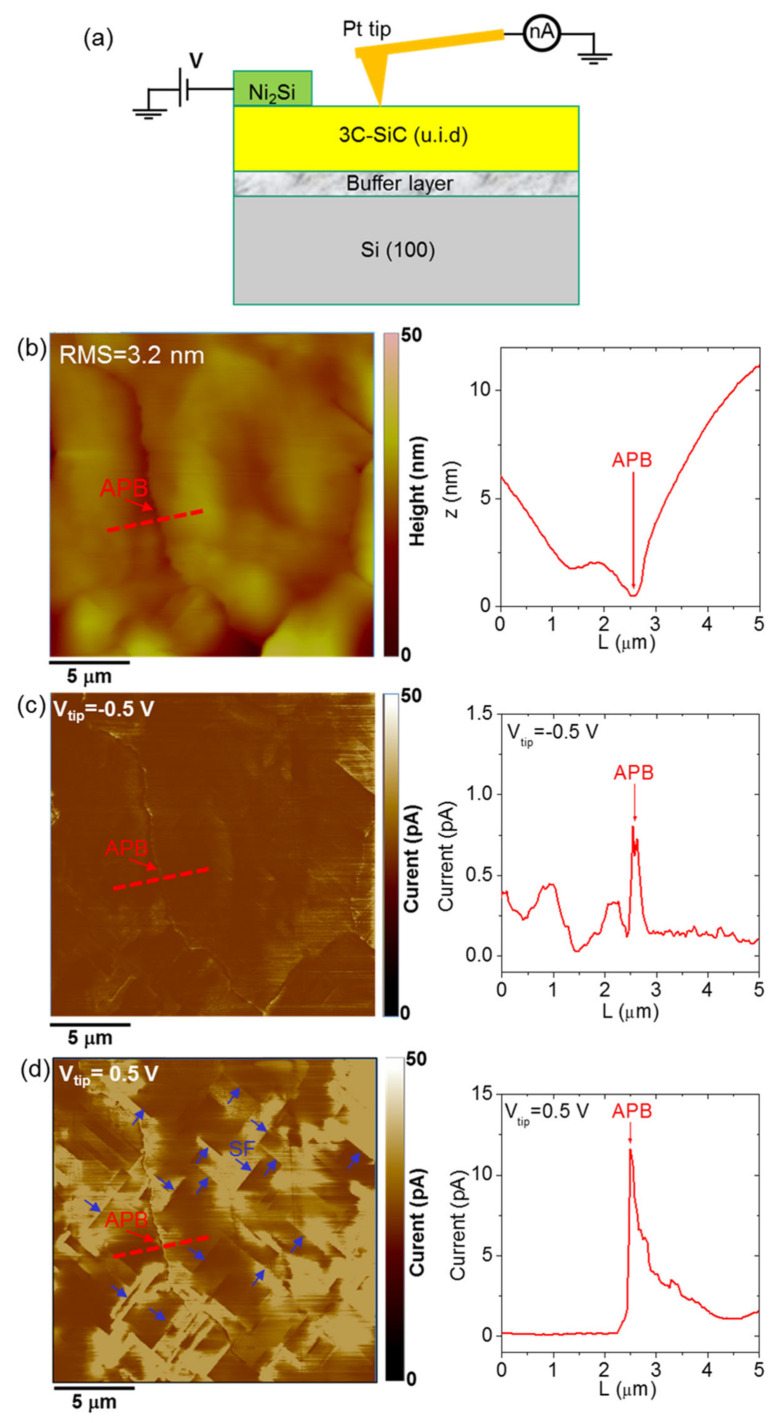
(**a**) Schematic illustration of the CAFM setup. (**b**) Morphology and (**c**) current maps collected under reverse-bias polarization of the tip (Vtip = −0.5 V) and (**d**) forward-bias polarization (Vtip = 0.5 V). An APB is indicated by a red arrow and SFs by blue arrows. Representative line-scans across a grain boundary extracted from the topography ((**b**), right panel), current maps under reverse-bias polarization ((**c**), right panel), and forward-bias polarization ((**d**), right panel) of the tip are shown (see Reference [63]).

Nanoscale-resolution current mapping of 3C-SiC by conductive atomic force microscopy (CAFM) provided a direct demonstration of APBs as the main extended defects responsible for the enhanced leakage under reverse-bias, whereas both APBs and SFs were shown to act as current paths under forward polarization. Figure 16a illustrates the experimental configuration used for CAFM measurements on the cubic silicon carbide surface. A typical topographic image collected on a 20 μm × 20 μm scan area is shown in Figure 16b, from which a surface root mean square (RMS) roughness of 3.2 nm was calculated. The nanometer deep “V-shape” depression in the morphology and in the height line-scan (Figure 16b, right panel) were associated to an APB in accordance with the Monte Carlo simulations of Section 3.7. Figure 16c,d report the current maps measured simultaneously to the topography by applying a reverse-bias (V_tip_ = −0.5 V) and forward-bias (V_tip_ = 0.5 V) polarization to the Pt tip, respectively. This Schottky diode behavior of the Pt/3C-SiC contact was confirmed by the significantly lower current values measured under reverse polarization with respect to those measured under forward-bias. Using the same current range (from 0 to 50 pA) for the two current maps, APBs are the most evident conductive features under reverse-bias, whereas both APBs and SFs (indicated by blue arrows in Figure 16d) contribute to the conduction under forward polarization. Two representative scan lines across the APB for the two opposite tip biases are also shown in the right panels of Figure 16c,d, showing a higher current peak on the APB under forward-bias with respect to the reverse one. This suggests that APBs are mainly responsible for the enhanced reverse leakage current measured in macroscopic Pt/3C-SiC Schottky diodes. In particular, the separation between these extended defects deduced from this microscopic analysis is in the order of tens of micrometers, in very close agreement with the value of L (≈20 μm) deduced from the statistical characterization of Schottky diodes with different areas for thin 3C-SiC layers [63].

### 3.11. Defects in 3C-SiC: Point Defects

Point defects can be observed in 3C-SiC by PL measurements on different samples grown in different conditions. In particular, the PL spectra in the wavelength range of 1100–1600 nm of different samples grown at different temperatures with the same growth rate are reported in Reference [64]. It was possible to observe that carbon vacancy (V_C_), carbon–silicon vacancy (V_C_V_Si_), carbon vacancy–Si antisite (V_C_C_Si_), and Al-related defects are present [65]. The growth rate and growth temperature seem to be the main parameters that influence the point defect formation during the growth, as also reported in a previous simulation paper [66].

### 3.12. Defects in 3C-SiC: Protrusions

**Figure 17:**
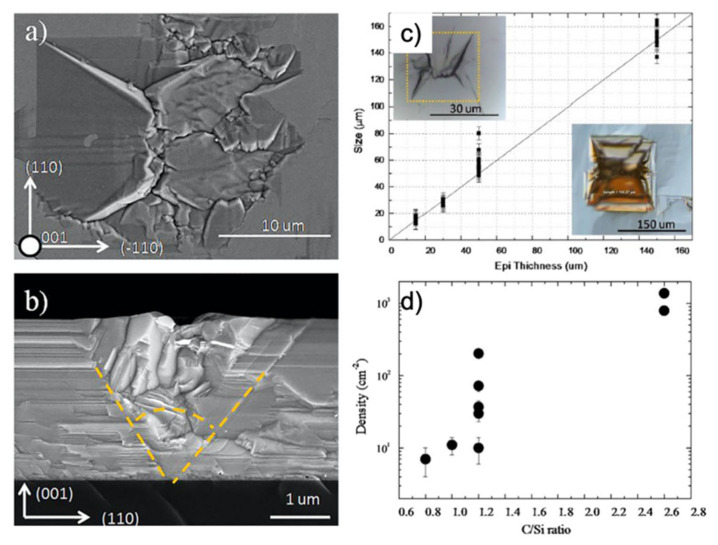
(**a**) Scanning electron microscope plan-view image of a protrusion in a 30 μm-thick epitaxial layer. (**b**) Cross-view obtained after the cleavage of the wafer for a 3 μm-thick epilayer. Yellow lines are drawn in order to identify the edge of the defect. Crystallographic orientations are also drawn. (**c**) The average size of the protrusions vs. the epitaxial layer thickness. (**d**) The density of the protrusion as a function of the C/Si ratio during the buffer layer step (adapted from Reference [67]).

Small defects produced during the carbonization process can have a large influence on the final wafer quality: an example of this effect can be seen in the case of the defect called “protrusions”. These defects appear on the surface of 3C-SiC as dark squares, with a peculiar 3D structure similar to inverted pyramids with a vertex close to the SiC/Si interface. In Figure 17a,b, a scanning electron microscope image of protrusion in in-plane (a) and in cross-view (b) are shown. [67] The plan-view image is shown for a 30 μm-thick epilayer to evidence the shape of the structure, while the cross-view is shown for a 3 μm-thick epilayer. Yellow lines are drawn to identify the edge of the defect that is limited by four stacking faults. It was also found that the inner core of the defect consists of nano-crystals twinned with respect to the substrate orientation. The base of the inverted pyramid is a square and the height is the same as the thickness of the epilayer. In Figure 17c, the lateral size of the protrusion is shown as a function of the epilayer thickness. A linear correlation between the size of the protrusion and the thickness of the epilayer is apparent. Again in Figure 17c, two optical images of the protrusion are shown: in the left-upper corner, a defect in the 30 μm-thin film is shown, similar to what was observed in Figure 17a, while in the right-lower corner, the defect in the 150 μm-thick film is shown. Since the quality of the layer depends strongly on the thickness, a higher thickness is preferable. Thus, the presence of even a small density of protrusions must be indeed avoided because it can strongly decrease the quality of the wafer. In stating the importance of reducing the density of such a defect, our group investigated the reason for the formation of protrusions. The seed of this extended 3D defect lies 10 nm above the SiC/Si interface and is probably related to a non-balanced carbon amount during the carbonization step or during the temperature ramp-up after carbonization. Carbonization, as earlier reported, is a process in which the carbon precursor reacts with the bare silicon surface and this is performed to prepare the Si surface for SiC growth. The Si/C ratio during the carbonization and the post-carbonization process is the key parameter to avoid the formation of protrusions. In Figure 17d, the density of protrusions as a function of the C/Si ratio during the rise of the temperature after carbonization is shown. As it is apparent, a ratio lower than 1.2 is able to decrease the density of protrusions by almost two orders of magnitude, leading to a density of 10 cm^−2^.

### 3.13. Stress in 3C-SiC

Another aspect that is crucial in the development of the 3C-SiC material is stress. In this case, we have essentially two different components of the stress:. The first one is called intrinsic stress, related to the different lattice constant between 3C-SiC and the silicon that produces a high concentration of defects at the interface. These defects produce a high level of stress essentially in the first microns of the growth. For thick layers, we observe a reduction of this intrinsic stress and for very thick layers, this component of the stress is close to zero. For the intrinsic stress, it has been observed through using MEMS devices that the reduction of the stress follows an exponential low that is very close to the exponential decrease of the SFs’ density vs. thickness [9]. This stress is generally compressive in 3C-SiC (100) while it is tensile in 3C-SiC (111).

The second component of the stress is the “thermal stress”, related essentially to the different thermal coefficients between silicon carbide and silicon. In fact, the growth occurs at high temperatures (1350–1390 °C) and the two materials (SiC and Si) decrease their lattice constants in different ways, moving from the growth temperature to room temperature. This component of the stress is always tensile and then reduces the stress or even changes the sign of the stress in the (100) material, while considerably increasing the total stress in the case of the (111) 3C-SiC. For this reason, it is extremely difficult to grow a thick layer of 3C-SiC on the (111) Si without cracking the film or even the substrate during the ramping down of the temperature in the reactor.

In the CHALLENGE project, we used two different approaches to try to solve this problem. The first approach has been described in Section 3.2. In fact, using the pillars’ structures, it is possible to considerably reduce the thermal stress with the deflections of the pillars on the edge of different patches (see Figure 7). In this way, it was possible to obtain thick (111) 3C-SiC layers’ wafers with a low bow.

The other approach that we used in this project will be described later in Section 3.15. In this approach, after a thick growth (60–90 μm), the silicon substrate was melted inside the reaction chamber and then removed one of the sources of the thermal stress. Obviously, this process does not remove the intrinsic stress due to the defects at the 3C-SiC/Si interface. Using the SiGe buffer layer approach described in Section 3.3, it is possible to decrease the intrinsic stress but further experiments should be done to completely remove this component.

### 3.14. Bulk Growth on Hexagonal SiC

**Figure 18:**
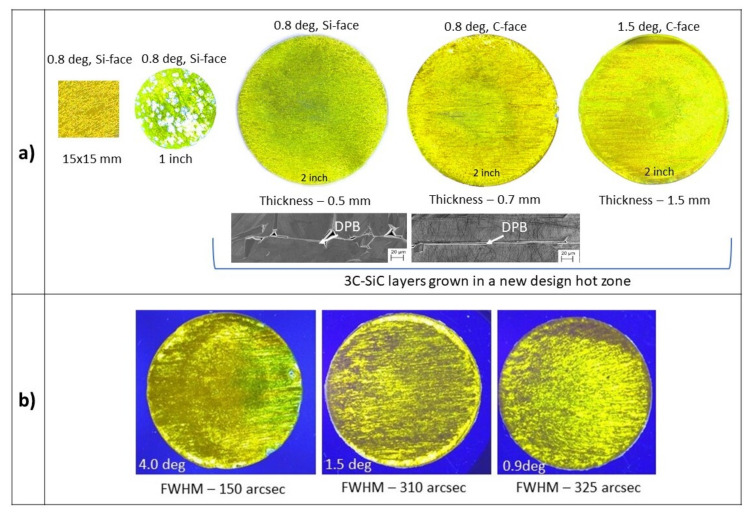
(**a**) 3C-SiC growth on hexagonal SiC substrates using enhanced sublimation epitaxy. (**b**) Polarized light optical micrographs of 2.5 mm-thick 3C-SiC layers grown on 2-inch 4.0, 1.5, and 0.9 degrees off-oriented SiC (000-1) substrates. All samples were grown at 1950 °C in vacuum (5 ×10^-4^ mbar).

As demonstrated [21,22], during the initial stages of the growth, a facet with an on-axis surface is formed at the edge of the grown layer. At specific growth conditions, this facet becomes a preferential 3C-SiC nucleation site. Once 3C-SiC is formed on the facet, it laterally enlarges by covering the entire surface. The lateral enlargement of 3C-SiC from the edge towards the center on the SiC (0001) surface is proportional to ~ tan α, where α is the off-cut angle of the substrate. Therefore, by increasing the off-orientation of the substrate, the total layer thickness needed to cover the entire substrate surface, with the 3C-SiC enlarging from the edge of the sample, also increases. Based on our estimations, to cover a 1-inch 4 degrees off-oriented hexagonal SiC (0001) substrate with 3C-SiC, the layer thickness should be about 4–5 mm. Such a thickness complicates the growth process. Therefore, as a compromise, a hexagonal substrate with a 0.8 degrees off-cut was used to explore the growth on a larger substrate area. As seen on the left side in Figure 18a, a full surface coverage with the 3C-SiC on hexagonal substrates with the size 15 × 15 mm^2^ was obtained. However, when the same growth conditions (T = 1900 °C, average growth rate of ~0.3 mm/h) were applied to the growth on 1-inch substrates, an instability of the 3C-SiC polytype was observed. This was attributed to the dimensional limitations of the graphite container, which does not allow for the obtaining of the uniform supersaturation of SiC vapor species over the entire surface of the substrate. Therefore, a new hot zone for the growth of 3C-SiC on 2-inch substrates was designed and the immediate advantage of it in controlling the stability of 3C-SiC was observed. However, by growing thicker layers on (0001)/Si-face substrates, it was observed that the DPBs tended to branch out into larger structural defects, which deteriorates the quality of the 3C-SiC crystal. To compare the formation of DPBs, the growth of 3C-SiC on the (000-1)/C-face was investigated. It was observed that there was an obvious difference in the DPBs’ appearance in the 3C-SiC grown on the (000-1)/C-face. As shown in the scanning electron microscope (SEM) images in Figure 18a, the majority of DPBs on the (000-1)/C-face maintain a line-like propagation path. This means that their propagation is less damaging to the 3C-SiC crystal compared to the ones on the Si-face. In addition, an interesting phenomenon indicating different step dynamics in 3C-SiC layers grown on the Si and C-faces has been observed. Surfaces analysis by atomic force microscope demonstrated that the step-height in both cases is very similar and mostly varies between 0.25 and 0.8 nm, while the terrace width is almost three times larger on the Si-face and varies in a range of ~130–150 nm. The crystalline quality of 3C-SiC layers grown on the Si and C-face on substrates with the same off-cut angle of 0.8 degrees is similar and the full-width at the half-maximum of the XRD ω rocking curve using a footprint of 5 × 5 mm^2^ varies between 200 and 300 arcsec. However, 3C-SiC layers grown on the C-face of a hexagonal substrate with the off-cut angle of 1.5 degrees contain areas with ω rocking curve values of 93 arcsec. This indicates that using hexagonal SiC substrates with even higher off-cut angles could be the right direction for further research, even though the growth on such substrates will require growing much thicker layers to obtain a full coverage with 3C-SiC. Therefore, a comparative study of the 3C-SiC(111) grown on the (0001)/Si-face and (000-1)/C-face on 4 degrees off-oriented 4H-SiC research size (7 × 7 mm^2^) substrates was conducted [68]. Even though the 3C-SiC polytype is more stable on the (0001)/Si-face, it was shown that smoother surfaces of 3C-SiC could be obtained on the C-face. In addition, the transition layer, which is a mixture of various polytypes, between the hexagonal SiC substrate and the 3C-SiC is significantly thinner on the (000-1)/C-face, leading to a direct polytype conversion mechanism.

Based on the promising results on the growth of 3C-SiC on the (000-1)/C-face of 4 degrees off-oriented 4H-SiC research size (7 × 7 mm^2^) substrates, a series of experiments were done on a 2-inch area. An example of a 2.5 mm-thick 3C-SiC layer grown on a 4 degrees off-oriented substrate is shown in Figure 18b (picture on the left). The 3C-SiC layer grown on 4 degrees off-oriented substrates still contains double-positioning boundaries that deteriorate the crystalline quality. Despite that, when compared to 2.5 mm-thick 3C-SiC layers grown on substrates with lower off-orientations, the crystalline quality is higher. This was confirmed by the FWHM of HRXRD ω rocking curves, which were measured on three different areas on each layer using a footprint of 2 × 10 mm^2^. The average FWHM values of 3C-SiC layers grown on 4.0, 1.5, and 0.9 degrees off-oriented SiC substrates were 150, 310, and 325 arcseconds, respectively. This indicates that even larger off-orientations could be a potential route for the further improvement of 3C-SiC crystalline quality.

**Figure 19:**
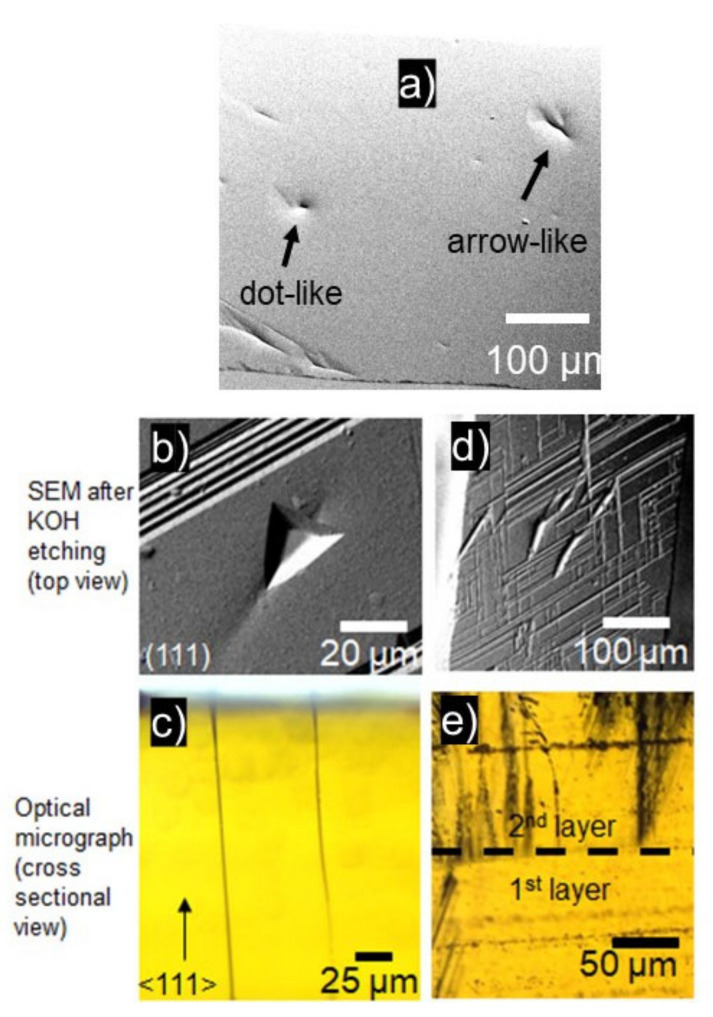
Top view SEM image after KOH etching of (**a**) threading dislocation and (**d**) arrow-like defects. Optical micrograph (cross-sectional view) of (**b**) threading dislocations and (**e**) arrow-like defects. (**c**) SEM image of the surface appearance of defects.

In addition to DPBs, which are dominant defects in 3C-SiC grown on hexagonal SiC substrates, dot-like and arrow-like defects are observed (Figure 19a). These defects usually occur in samples that are thick (>1 mm) and grown by interrupted growth (growth stopped to change polycrystalline SiC plate/source). After selective etching using molten KOH, the dot-like defects appear as triangular etch pits (Figure 19b), which are characteristic features of threading screw dislocations on the (111) crystal surfaces. The selective etching of arrow-like defects revealed elongated groves as shown in the SEM micrograph in Figure 19d. In addition, after KOH etching, the surface around the elongated grooves possessed imprints of stacking faults propagating along (111) planes, which appeared as line-like features rotated to each other by 60 degrees. Based on cross-sectional analysis by optical microscope, the dot-like features on the surface corresponded to the threading defects with a cylindrical path that did not widen while the crystal grew (Figure 19c). They originate in the grown 3C-SiC layer, transition layer, or can be tracked down all the way to the substrate. In the latter case, they can be observed as a continuation of micro-pipes propagating in the substrate material. The arrow-like defects can originate anywhere in the 3C-SiC layer but the most common origin is at the interface between 3C-SiC layers grown by repeated growth runs as shown in Figure 19e. This indicates that thick (>1 mm) 3C-SiC layers should be grown in a single growth run. Otherwise, disturbances in the growth during the cooling down and temperature ramp-up processes may cause the formation of such defects. Moreover, as the thickness of the 3C-SiC layer increases, the arrow-like defects tend to branch out and significantly deteriorate the crystal quality. The formation of such defects can be tackled by using thicker source-material, which would allow for growing thick layers without any interruption.

Even though the defect density in the 3C-SiC grown on hexagonal SiC over the 2-inch area is too high for the industrial processing of transistors, it could be used to explore hydrogen generation using solar-driven water splitting [69] or the growth of a large-area monolayer and multilayer graphene [70,71].

### 3.15. Close-Space PVT Growth of Bulk 3C-SiC on 3C-SiC-on-Si CVD Seeding Layers

**Figure 20:**
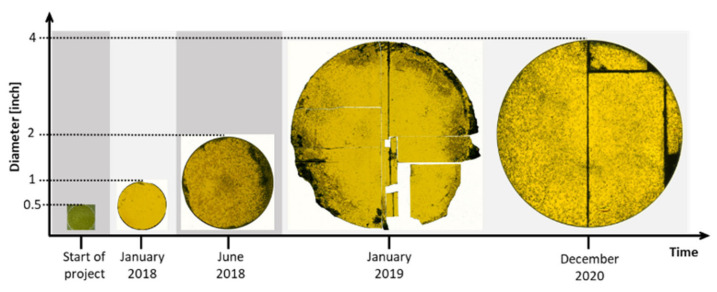
Evolution of diameters for bulk 3C-SiC crystals grown by sublimation growth. The timeline is indicated.

Figure 20 gives an overview of the development of the diameter of grown crystals during the project. Starting with a diameter of 0.5 inches at the beginning of the project, the first big milestone was reached in 2018 with the reproducible growth of 2-inch crystals with thicknesses of up to 870 µm. This marked the first time that bulk material, with relevant sizes, was grown regularly using a sublimation method [26]. Such a material could be used as a seed for subsequent growth in other processes such as M-PVT or CF-PVT. With the transfer process described in Section 2, crack-free crystals could only be obtained for up to 2 inches. For larger diameters, cracking of the thin epitaxial layer poses a problem during the preparation of the seeding stack. Nevertheless, the first growth runs on 4-inch materials were performed at the beginning of 2019. Additional improvements of the transfer process were necessary to optimize the results for the large sample size. Although the cracking problem could not be solved at this point, first-cracked but non-broken samples with a thickness of approximately 1 mm could be produced by the end of 2020, consisting of one coherent piece of crystal. Measurements of the XRD rocking curve of the full-width at half-maximum (FWHM) of the (002) reflex resulted in values of 138 and 140 arcsec for 2-inch and 4-inch materials, respectively, confirming that there is no decline in material quality for large diameters.

There are two main reasons for cracking during the manufacturing of seeding stacks. The first one is associated with the used starting material grown by CVD. Caused by a lattice mismatch between silicon and 3C-SiC of approximately 20%, a wafer bow occurs during the heteroepitaxial growth [11]. The bow and the accompanying stress in the material will increase the probability of cracking the thin 3C layers during handling. This problem will be even bigger with increasing diameters. The second issue occurs during the etching process. During this step, NO_x_ species will be created [36]. As the silicon removal starts at the edges and moves towards the center of the samples, the created NO_x_ species will accumulate in the middle of the sample, leading to the buoyancy of the thin remaining 3C layer. This mechanical stress will lead to cracks. For diameters up to 2 inches, this problem can be neglected but will be present for larger samples. One solution to prevent the uplift concerned the change from a horizontal to a near-vertical etching setup, reducing the effect of sample bending and therefore reducing the cracking probability. Nevertheless, micro defects induced at the edges during the laser ablation process, as well as the wafer bow, still lead to the cracking of the seeding layers.

Due to ongoing research, a new form of seeding material became available. As described by Anzalone et al. [64], the production of freestanding 3C-SiC wafers grown homoepitaxially by CVD at elevated temperatures is possible. The availability of such seeding materials, up to a thickness of approximately 200 µm, offers new possibilities for the continuing growth using CS-PVT. Compared to the thin epitaxial seeds, no transfer process is necessary for such materials. In addition, the material grown by homoepitaxial CVD still has some setbacks concerning the wafer bow and remaining protrusion defects, and first-successful sublimation growth runs on such seeds were carried out. These experiments have proven its suitability as a seed for CS-PVT and therefore represent a promising starting point for the bulk growth of cubic silicon carbide.

Despite the appearance of different defects on the surface, all samples depicted in Figure 20 have a bright yellow appearance typical for the cubic polytype. The results of X-ray diffraction (XRD) and Raman spectroscopy confirm the growth of 3C-SiC. The small black dots visible are associated with protrusion defects that were already present in the CVD seeding layers. These defects increase in size with the increasing layer thickness during the growth process. The darker areas at the edges of the samples were caused by both an overflow of the carbon glue during the merging step and an insufficient cleaning step afterward.

Besides the clearly visible defects, the material quality of the sublimation-grown crystal is quite high. Raman spectrometry is commonly used for the analysis of material quality as it provides a fast and non-destructive method for the evaluation of material quality. The transversal optical (TO) mode should be forbidden for defection-free (100)-oriented on-axis-grown cubic silicon carbide. However, if it is visible, it can be used to determine the stress inside the material, depending on the position of the peak [72,73]. For stress-free materials, the wavenumber of the peak is located at approximately 797.61 cm^−1^ [74]. An increased value is linked to comprehensive stress, whereas a lower value can be related to tensile stress. In [26], the wavenumbers for different types of cubic silicon carbide materials were presented. It could be shown that the layers grown by CVD were tensile-stressed, caused by the lattice mismatch between silicon and 3C-SiC both for on-axis as well as off-axis materials. The value for the crystals grown by the sublimation method, with CVD materials as seeds, displayed similar stress levels. After the sublimation growth, the crystals were usually oxidized at 800 °C to remove the carbon glue and separate the samples from the polycrystalline SiC-carrier. After this treatment, the measured values for the TO mode were near the stress-free value of 797.61 cm^-1^ as reported in the literature, indicating a quasi-stress-free material. In addition, XRD 2θ-ω scans were performed. For a stress-free cubic silicon carbide crystal, the in and out-of-plane lattice constant should be the same based on the cubic lattice. As stress will distort the lattice, a variation of the lattice planes will occur. The data obtained from the measurement confirm the assumption of the stress-free material after the sublimation growth and removal of the carrier. The results can be found in [33].

**Figure 21:**
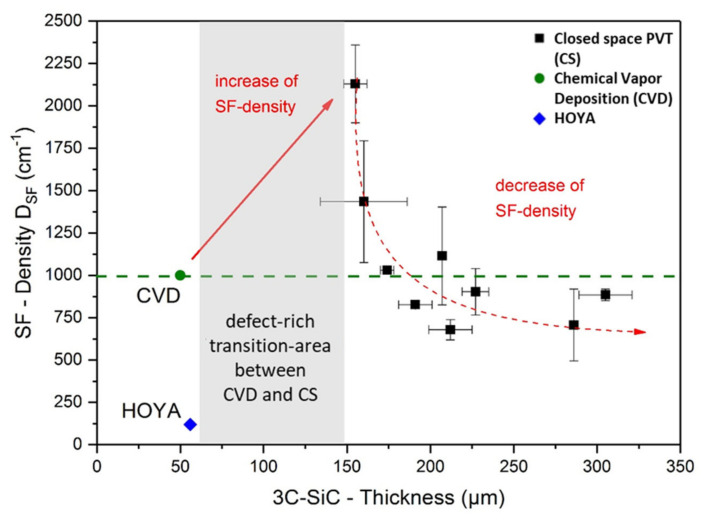
Stacking fault (SF) density of KOH-etched 3C-SiC samples with regard to grown layer thickness using CS-PVT. After an initial rise of SF density due to the defect-rich transition area between CVD and CS, the SF density will decrease with increasing 3C-SiC thickness and even with a value below the value of the used CVD seed. The SF density of the HOYA sample grown by switch-back-epitaxy is presented as a comparison. Adapted from [75].

Typical defects occurring in (100)-orientated 3C-SiC are stacking faults (SF), anti-phase boundaries (APB), and protrusions. To evaluate the evolution of SFs during CSPVT, KOH etching was performed. Depicted in Figure 21 is the SF density for hetero-epitaxially grown CVD material as well as the values for sublimation-grown samples with regard to their thickness. Starting from CVD seeding materials, a defect-rich transition layer will form between the seed and the sublimation-grown material, increasing the SF density. As the CS-PVT growth will continue, this density will decrease with the increasing layer thickness. The density saturates at a level lower than the compared CVD material if the layer thickness reaches a thickness of approximately 200 µm [75].

**Figure 22:**
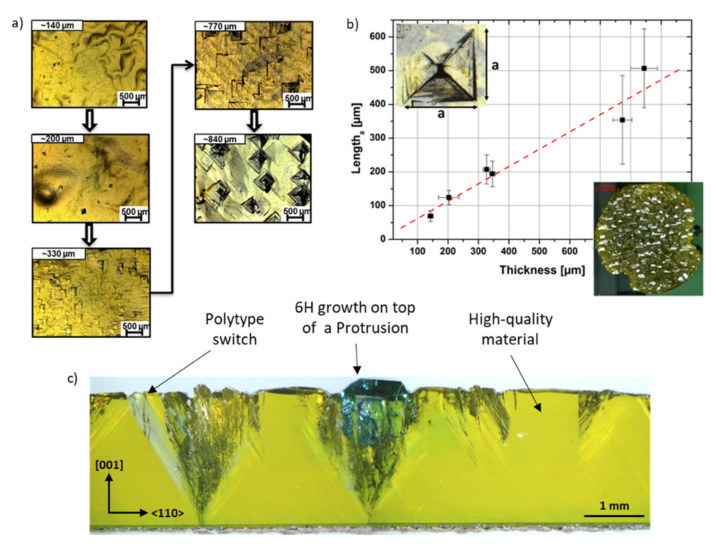
(**a**) Size of protrusion defects for bulk 3C-SiC layers with different 3C-SiC thickness. (**b**) Edge length of protrusions plotted versus 3C-SiC thickness. Additionally, the surface of an approximately 2.7 mm-thick grown crystal with a diameter of 25 mm is visible. The crystal is completely dominated with protrusion defects, leading to a ragged surface. (**c**) Cross-cut of a 3.4 mm-thick crystal revealing polytype switches as well as the inner parts of the protrusion defect. Areas between and underneath the protrusions show high quality material grown by CS-PVT.

The characterization of the material using Raman, XRD, and KOH-etching for the evaluation of the SF density shows an improvement in the material quality for CS-PVT compared to the seeding material grown by heteroepitaxial CVD. However, the most important value regarding the real bulk growth is the thickness of the grown crystals. The limitations of the achievable thickness to this point are strongly connected to the type of defect in the material. Protrusion defects were already present in the seeding layers and have their origin in the carbonization step during CVD growth. These three-dimensional defects tended to increase with increasing layer thickness as observed by Zimbone et al. [67] during subsequent CVD growth. In starting with such materials as a seed, a similar trend could be observed for CS-PVT [75]. Optical images of the “as grown” surface for a set of growth runs are depicted in Figure 22. It is clearly visible that the size of protrusions increased with the increasing layer thicknesses. At least for the investigated parameter range, this trend can be described as linear. Consequently, for even large thicknesses (d > 1 mm), the surface became more and more dominated by these defects. For bulk, growth runs with a thickness of approximately 3 mm, with a rough surface completely covered with protrusions, was observed (Figure 22b). In addition to the ragging of the surface, polytype switches towards 6H-SiC can be observed in the crosscut depicted in Figure 22c and can be found near or on top of protrusion defects. The edges of the defects are formed by stacking faults in the (111) planes. As these planes were equivalent to the (0001) faces in the hexagonal system, the probability for the nucleation of 6H-SiC could increase at these sides. Additionally, the growth of 6H-SiC on top of protrusion defects seems to confirm the loss of nucleation information for the continuing growth of the cubic polytype.

The reduction of protrusion density in the CVD seeding layers is an important task towards the real bulk growth using sublimation growth, especially as the material grown by CS-PVT shows a very high quality in the areas in which no protrusion defect is present. Therefore, efforts were made to reduce the protrusion density during the sublimation growth. One approach features the growth on the original transition layer from the CVD growth between the silicon and the cubic silicon carbide. Schuh et al. [76] showed that a slight reduction of the protrusion density could be observed using this transition layer as a starting point for the sublimation growth instead of the original CVD growth front. During regular CS-PVT, a partial overgrowth of protrusion defects could also be observed, as depicted in [77]. This effect could be observed for growth runs on on-axis seeding material as well as for 4° off-orientated-grown seeds. So far, the mechanism behind this overgrowth is not completely understood. It seems that this effect is more pronounced for off-axis-grown samples compared to on-axis grown samples.

### 3.16. Simulation of PVT Bulk Growth

To determine the growth conditions present inside the growth cell during the sublimation growth of 3C-SiC, numerical modeling of the temperature field and mass transport-related phenomena were performed. The basic aim was to first identify the growth conditions existing in the 50 mm apparatus and then to use such data to ensure stable growth conditions to enlarge wafer sizes of 100 mm and greater. The study examined the effect of the appropriate SiC characteristics and the various carbon materials serving as process values in the computational modeling for both the temperature profile and the associated mass transport. In order to implement the thermal field and mass transport effects, computer simulation was performed through COMSOL Multiphysics. The appropriate selection of the physical parameters related to graphite-based components, as well as for the carbon isolation characteristics over 2000 °C, appeared to not be an easy task. The main issue concerns the ambiguity, if not a complete absence of reliable data, on the temperature behavior of electrical and thermal conductivity at the growth thermal conditions.

Nonetheless, numerical modeling allows for not only the calculation of the small growth cell but also the simulation of the whole growth reactor. Besides the calculation of temperature fields, mass transport, and supersaturation, simulation with COMSOL Multiphysics provided insight into the behavior of magnetic fields as well as into the formation of hot zones.

Worthy to note, despite the fact that there is no unambiguous data on the behavior of thermal conductivity at higher temperatures for graphite crucibles and insulating components, it is possible to assert using calculations and experimental calibrations that the thermal gradient in the gas phase has no effect, unless for the second order of approximation. As a consequence, it is expected that calculating, for example, supersaturation in front of the growth interface would offer accurate findings useful for the design and optimization of growth cells.

**Figure 23:**
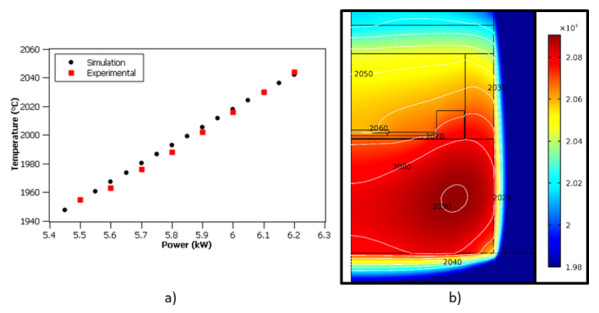
(**a**) Comparisons between simulations using COMSOL Multiphysics and the measured temperatures at the crucible top during growth runs for different heating powers in the 50 mm-CS-PVT setup. (**b**) Typical temperature field for CS-PVT. Some isotherms are indicated with the corresponding temperatures.

The supersaturation of the SiC_2_ gas species plays an important role, influencing the growth-limiting parameter at the seed-growth front. This supersaturation can be calculated using the partial pressure of the gas species at the seed and source. Different approaches for the calculation of the supersaturation can be found in literature, for example, by Lilov [78] or Avrov [79]. Each experiment is similar in that for the calculations, the knowledge of the actual temperatures during the growth process is required. A comparison between the simulation data and the measured temperatures for the 50 mm-CS-PVT growth setup at different heating powers, showing a good agreement, is depicted in Figure 23a. A similar trend could be observed for the 100 mm-growth cell. Additionally, an example of the temperature field present in the growth setup can be seen in Figure 23b.

Using the temperatures obtained at the seed and source from the simulations, the supersaturations present during the sublimation growth can be calculated using the equation mentioned by Rankl et al. [80]. They found that for the heteroepitaxial growth of 3C-SiC on (0001)-oriented 6H—SiC, a supersaturation as high as s = 0.4 is necessary to achieve a high yield. Based on the development regarding the carbon materials databases, this value was revised to s = 0.24 [32]. In the case of homoepitaxial sublimation growth on seeds already containing the cubic polytype, a supersaturation higher than s = 0.1 was found to be suitable to ensure stable growth. It was also found that for this purpose, a source to seed a distance of 1 mm or smaller is necessary depending on the growth temperature as the supersaturation will decrease with the increasing spacing [31].

A global model for the evaluation of the processes’ results in terms of the material growth rate can be obtained from the estimates of the mass transfer rate from the seed to the substrate once the temperature field is evaluated by the chamber simulation, as discussed in the previous section. Assuming that ballistic transport conditions occur for the Si-C molecules, which sublimate at different rates at the two interfaces, approximate estimates of the growth rate for the 3C-SiC in the different positions of the growing substrate (in a fully symmetric configuration) can be obtained from the balance between the atomic species’ effective deposition fluxes (j_dep_), derived from the sources and ruled by the source temperature, and the evaporation flux (j_ev_), ruled by the substrate local temperature. Due to the composition of the SiC vapor pressure, Si-rich conditions are usually assumed (see Avrov et al. [78]) and the growth rate can be estimated by:

(1) $$Gr=\frac{\rho_{SiC}}{M_{SiC}}\left(j_{dep}-j_{ev}\right)Gr$$

using the atomic carbon effective flux only. In Equation (1), $\rho_{SiC}$ is the SiC density and $M_{SiC}$ is the SiC molar mass. The expression for $j_{dep}$ and $j_{ev}$ are given by

(2) $$j_{dep}=j_{dep}\left(C\right)=j_{dep}\left(Si_{2}C\right)+2j_{dep}\left(SiC_{2}\right)\;\;=\sqrt{\frac{2\pi RT_{Source}}{M_{Si_{2}C}}}\exp\left(\frac{A_{Si_{2}C}}{T_{Source}}+B_{Si_{2}C}\right)+2\sqrt{\frac{2\pi RT_{Source}}{M_{SiC_{2}}}}\exp\left(\frac{A_{SiC_{2}}}{T_{Source}}+B_{SiC_{2}}\right)$$

(3) $$j_{ev}=j_{ev}\left(C\right)=j_{ev}\left(Si_{2}C\right)+2j_{ev}\left(SiC_{2}\right)\;\;=\sqrt{\frac{2\pi RT_{Sub}}{M_{Si_{2}C}}}\exp\left(\frac{A_{Si_{2}C}}{T_{Sub}}+B_{Si_{2}C}\right)+2\sqrt{\frac{2\pi RT_{Sub}}{M_{SiC_{2}}}}\exp\left(\frac{A_{SiC_{2}}}{T_{Sub}}+B_{SiC_{2}}\right)$$

where $T_{Source}$ and $T_{Sub}$ are the source and the substrate temperatures; $M_{Si_{2}C}$ and $M_{SiC_{2}}$ are the molar masses of the *Si_2_C* and *SiC_2_* molecules in the vapor phase; and *A_x_* and *B_x_* are the experimental parameters that rule the partial pressures of the *X* species in the vapor mixtures at the thermodynamic equilibrium with the solid counterpart. Different calibrations for the partial pressures-related parameters can be found in the literature and by using the one in Reference [78], a quantitative estimation of the growth rate in accordance with the experimental results can be obtained.

### 3.17. Bulk Growth by CVD

**Figure 24:**
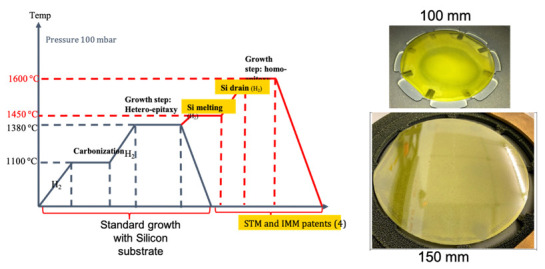
Schematic of the new process for CVD bulk growth (left). 3C-SiC wafers with the dimensions of 100 mm and 150 mm grown with the new process (right, adapted from Reference [64]).

To obtain a 3C-SiC bulk material of up to 6 inches (or 8 inch in the future), a new epitaxial reactor chamber was designed and tested in several experiments. The major idea was to hetero-epitaxially grow on silicon a 3C-SiC layer and use it as seeds, melt the silicon substrate, and again start the growth at very high temperatures. Thereby, we grew a bulk substrate of 3C-SiC with a low density of SFs and low wafer bow. Most importantly, the bow can be strongly decreased since by removing silicon, the stress due to the different thermal expansion coefficients between the two materials is removed. The silicon melting provides an increase in the growth temperature and in the growth rate. In this way, thicker wafers and better crystal quality of the material can be achieved. For a better understanding of how to melt the silicon substrate, how to drain it, how to etch the remaining silicon, and how to grow the homoepitaxial layer on the 3C-SiC substrate obtained after the silicon melting, many experiments have been performed. In Figure 24 (left), a scheme of the entire process is reported. The first two steps are the standard ones (well-reported and described in the literature).

After the melting, the SiC layer was used as a seed layer for subsequent homoepitaxial growth. [64] In this way, growing both 100 mm and 150 mm wafers as reported in Figure 24 (right) was possible.

**Figure 25:**
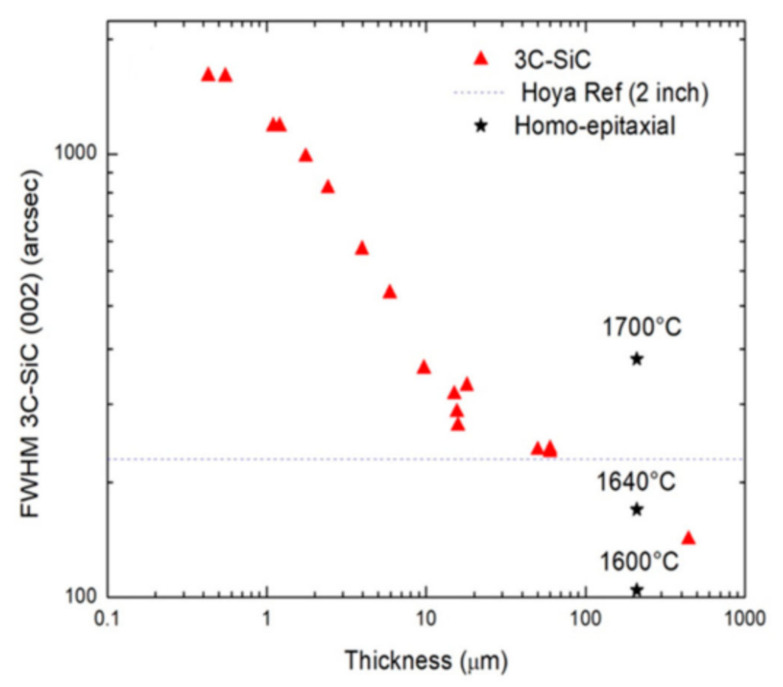
FWHM of the 3C-SiC (002) peak vs. the grown thickness. The decrease of the FWHM at high 3C-SiC thickness can be observed. The low temperature growth shows a much better quality of the material (see Reference [64]).

The effect of temperature on the homo-epitaxial process was observed by X-ray diffraction analysis (Figure 25). The full-width of the half-maximum on the X-ray rocking curve of the 3C-SiC (002) peak was correlated to the crystal structure and defect density (lower FWHM value means better crystal quality). The figure shows the FWHM as a function of the film thickness for several samples. The samples reported in the graph were grown in different conditions (all the samples at a low value of thickness derive from previous experiments [6]). An increase in the film thickness has the effect of the quality of the material increasing [81]. The initial part of the curve (from 0 to about 20 µm of thickness) shows the 3C-SiC sample’s growth with the Si substrate. For such samples, the crystal quality was limited by the presence of the silicon substrate that, starting from about 20 microns of thickness, led to cracks and extended defects. These defects are generated during the cooling down process after the growth [6]. The three points between 60 μm and 90 μm are the 3C-SiC samples for which the silicon substrate was melted (Si fusion) and showed good crystal quality (around 200 arcsec), similar to the old 2 inch-3C-SiC wafer provided by the Hoya corporation (dotted line). These wafers were used as a template for the homoepitaxial process. The stared points (three at 200 micron) are the samples growth by using the new melting process explained in the current manuscript. They were grown at three different temperatures (1600 °C, 1640 °C, and 1700 °C). The sample grown at 1600 °C is appreciably better than the other two [64]. The FWHM value at around 100 arcsec is very promising also compared with a thicker sample (about 400 micron) grown at a high temperature by sublimation epitaxy (PVT reactor) [31].

## 4. Conclusions

In the CHALLENGE project, two main different approaches were used to grow 3C-SiC. From one hand, several compliance substrates (pillars, SiGe buffer layer, ISP, etc.) were used to reduce both defects and stress. On the other hand, new bulk growth techniques (PVT or CVD) have been developed to improve the quality of this material. During this work, a new understanding of the defects in this material (protrusions, APBs, SFs, and point defects), their interactions, and the effect of the growth process on their formation and reduction have been obtained. This new understanding has been also helped by the simulation codes developed inside the project (KMCsL, MD, phase field, etc.) and by new characterization techniques (C-AFM).

From this large study on the growth of 3C-SiC, several conclusions can be reported:

- The use of the SiGe buffer layer is interesting and in some cases, some good quality samples can be obtained. The main limitation is that a low-temperature growth should be used and the Ge segregation at the 3C-SiC/Si interface can produce the formation of polycrystalline regions. The process window is narrow and thus this process can be difficult to use in a production line.
- The ISP substrate produces a fast decrease of the SFs’ density, as reported in the previous papers, but at the same time produces the formation of APBs that can be detrimental for the leakage current of the devices, as observed by C-AFM measurements. To decrease the APBs concentration, a very high thickness can be grown or new ISP structures should be realized.
- The pillar substrates have demonstrated to be extremely interesting in reducing the stress and bow of the wafer, especially for (111) substrates. On this kind of substrate, even 25–30 μm of the 3C-SiC layer could be grown without cracks and with a low bow. The main problem of this process is that the 3D growth of 3C-SiC is not easy to control and thick layers are needed to obtain a continuous substrate. This kind of technology can be interesting in the future when a good 3C-SiC (111) layer should be realized.
- During the last years, we developed a carbonization process that decreases the voids almost to zero at the 3C-SiC/Si interface with a considerable improvement of the material quality and a decrease of the stress. Even the growth during the temperature ramp (buffer layer) between the carbonization and growth steps has a large effect on the stress.
- The main part of the investigation of the last years has been on the study of the evolution of SFs and APBs, as well as their interactions. The different types of SFs (SF<1>, SF<2>, SF<3>), their generation and annihilation, and the influence of the PD on these processes have been observed and studied in great detail. Another aspect that has been studied in detail is the interaction between the APBs and SFs. In fact, APBs can both generate or annihilate SFs and all these processes have been studied both experimentally and by simulations.
- It has been observed that due to this equilibrium between the generation and recombination of SFs, very thick layers are needed to obtain a low value of SFs that can provide the opportunity to realize a good device on 3C-SiC. The growth conditions (growth temperature, growth rate, and doping) can influence this equilibrium between generation and annihilation. It seems also that the different types of SFs have different behaviors during the growth but more investigations on this aspect are needed.
- From these studies, it has been understood that the bulk growth of 3C-SiC is needed to obtain a low value of defects compatible with the realization of power devices. We have developed different kinds of growth techniques (CVD and PVT) to realize bulk wafers with dimensions compatible with the actual standards (100 and 150 mm) but the main problem is related to the formation of the protrusions in the first instant of the growth. This 3D defect can grow and cover the entire surface of the ingot, thus a new process that can eliminate the formation of these defects is needed to obtain a 3C-SiC wafer for the realization of power devices.
- Another aspect of the 3C-SiC bulk growth that needs further investigation is the intrinsic stress of this material. In fact, the high level of stress and bow of the wafers are a problem for the further processing of the 3C-SiC wafers.

Further work should be done to obtain a good material for power devices, but the work performed during this project is a fundamental step for developing new materials for power devices.

## Data Availability

The data can be made available, upon reasonable request, asking to the corresponding author.
